# Alleviation of extensive visual pathway dysfunction by a remyelinating drug in a chronic mouse model of multiple sclerosis

**DOI:** 10.1111/bpa.12930

**Published:** 2021-01-29

**Authors:** Maria T. Sekyi, Kelli Lauderdale, Kelley C. Atkinson, Batis Golestany, Hawra Karim, Micah Feri, Joselyn S. Soto, Cobi Diaz, Sung Hoon Kim, Marianne Cilluffo, Steven Nusinowitz, John A. Katzenellenbogen, Seema K. Tiwari‐Woodruff

**Affiliations:** ^1^ Division of Biomedical Sciences Riverside School of Medicine University of California Riverside CA USA; ^2^ Department of Bioengineering Riverside Bourns School of Engineering University of California Riverside CA USA; ^3^ Department of Chemistry and Cancer Center University of Illinois at Urbana‐Champaign Urbana IL USA; ^4^ BRI Electron Microscopy Laboratory Los Angeles School of Medicine University of California Los Angeles CA USA; ^5^ Stein Eye Institute Los Angeles School of Medicine University of California Los Angeles CA USA

**Keywords:** demyelination, electroretinogram, estrogen receptor β, experimental autoimmune encephalomyelitis, indazole chloride, inflammation, multiple sclerosis, myelin, neurodegeneration, optical coherence tomography, remyelination, visual dysfunction, visual pathway, visually evoked potentials

## Abstract

Visual deficits are among the most prevalent symptoms in patients with multiple sclerosis (MS). To understand deficits in the visual pathway during MS and potential treatment effects, we used experimental autoimmune encephalomyelitis (EAE), the most commonly used animal model of MS. The afferent visual pathway was assessed in vivo using optical coherence tomography (OCT), electroretinography (ERG), and visually evoked cortical potentials (VEPs). Inflammation, demyelination, and neurodegeneration were examined by immunohistochemistry ex vivo. In addition, an immunomodulatory, remyelinating agent, the estrogen receptor β ligand chloroindazole (IndCl), was tested for its therapeutic potential in the visual pathway. EAE produced functional deficits in visual system electrophysiology, including suppression of ERG and VEP waveform amplitudes and increased signal latencies. Therapeutic IndCl rescued overall visual system latency by VEP but had little impact on amplitude or ERG findings relative to vehicle. Faster VEP conduction in IndCl‐treated mice was associated with enhanced myelin basic protein signal in all visual system structures examined. IndCl preserved retinal ganglion cells (RGCs) and oligodendrocyte density in the prechiasmatic white matter, but similar retinal nerve fiber layer thinning by OCT was noted in vehicle and IndCl‐treated mice. Although IndCl differentially attenuated leukocyte and astrocyte staining signal throughout the structures analyzed, axolemmal varicosities were observed in all visual fiber tracts of mice with EAE irrespective of treatment, suggesting impaired axonal energy homeostasis. These data support incomplete functional recovery of VEP amplitude with IndCl, as fiber tracts displayed persistent axon pathology despite remyelination‐induced decreases in latencies, evidenced by reduced optic nerve g‐ratio in IndCl‐treated mice. Although additional studies are required, these findings demonstrate the dynamics of visual pathway dysfunction and disability during EAE, along with the importance of early treatment to mitigate EAE‐induced axon damage.

## Graphical abstract

To understand deficits in the visual pathway during MS and potential treatment effects, the most commonly used animal model of MS, experimental autoimmune encephalomyelitis (EAE), was used. EAE‐induced retinal ganglion cell loss, axon demyelination, and VEP related increased latency were mitigated by the remyelinating drug indazole chloride. Measuring visual function and recovery in the presence of novel therapies can be used to screen more effective therapies that will protect axons, stimulate axon remyelination and prevent ongoing axon damage.

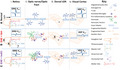

## 1 INTRODUCTION

The visual pathway is highly myelinated and particularly susceptible to the inflammatory demyelination prevalent in multiple sclerosis (MS). Visual deficits are often the initial presenting sign of MS and persist in almost all patients throughout the progression of the disease (1, 2). Visual deterioration is particularly devastating to MS patients, who consider it the second most valuable bodily function at risk after motor function in MS (3, 4).

The visual system can be segregated into the afferent and efferent visual pathways, both of which are affected in MS. The afferent system is the primary route by which visual stimuli are received and processed by the eye and brain. Visual manifestations of MS pathology related to the afferent visual pathway can include visual blurring as a result of increased body temperature or Uhthoff’s phenomenon, as well as impairments in motion perception, contrast sensitivity, low‐contrast letter acuity, and color discrimination (1, 4, 5, 6). In contrast, the efferent system is comprised of a series of CNS circuits which modulate and employ input from the afferent system to control secondary events such as oculomotor function and circadian responses. Efferent visual pathway dysfunction in MS can include ocular motility disorders, diplopia (double vision) and/or oscillopsia (perceived oscillation of objects in the visual field) (1, 7, 8).

Of the many visual pathologies associated with or predating MS diagnosis (1, 9, 10, 11), optic neuritis (ON), inflammatory demyelination of the optic nerve, is the most common. Approximately 50% of MS patients experience ON prior to exhibiting initial symptoms, while 70% develop the disorder at some point during MS disease progression (12). In addition, ON is an early MS event in most cases, even before motor or cognitive deficits are detected (9, 11). ON‐associated visual deficits include retinal nerve fiber layer (RNFL) thinning, loss of retinal ganglion cells (RGCs), and increased latency in visual evoked potentials (VEPs) (6, 13, 14).

Although the majority of visual deficit studies in MS and mouse models of MS have focused on the retina and optic nerves (15, 16, 17, 18, 19), posterior visual pathway structures such as the lateral geniculate nucleus (LGN), optic radiations (OR), and visual cortex are also significantly affected in MS independent of ON (13, 20, 21, 22). Specifically, MRI of the visual pathway in MS patients shows decreases in the volume of the LGN, lesions in the OR, and atrophy of the visual cortex (13, 20, 21, 22, 23). Even in the absence of OR lesions, significant increases in OR axial diffusivity (thought to be indicative of myelin damage), are often evident in MS patients compared to non‐MS controls (24). Functional assessment of the visual pathway in MS patients using VEPs reveals latency changes and amplitude deficits indicative of myelin loss and axonal damage (25, 26, 27). Due to this, a rigorous pathological and functional analysis of both anterior and posterior portions of the afferent visual system in MS and mouse models of MS is warranted. Using mouse models of MS to assess the visual pathway longitudinally will allow for understanding the disease mechanistically and finding the potential therapeutic targets.

Experimental autoimmune encephalomyelitis (EAE) is an animal model of MS that recapitulates autoimmune demyelination and neurodegeneration observed in patients with MS (28, 29, 30, 31). In the current work, the pathological and functional consequences of EAE‐induced inflammation and demyelination in the afferent visual pathway were investigated in the retina, optic nerve, optic tract, dorsal (d) LGN, and visual cortex. Optical coherence tomography (OCT) analysis of the retina, functional electroretinograms (ERG), visually evoked potential (VEPs) assessments, and immunohistochemistry (IHC) across the visual system were employed to assess the functional and structural changes.

Several studies have demonstrated the neuroprotective and therapeutic benefit of estrogen‐based treatments, which have been shown to reduce the clinical signs and the inflammatory lesions in EAE (32, 33). However, there are significant side effects associated with estrogen treatment, including feminizing effects in males and increased risk for breast and uterine endometrial cancer (34). These deleterious effects appear to be mediated primarily through estrogen receptor α (ERα) while estrogen receptor β (ERβ) is associated with tumor suppression and reduced cell proliferation (35). The ERβ agonist 3‐chloro‐2‐(4‐hydroxyphenyl)‐2H‐indazol‐5‐ol (IndCl) developed by the Katzenellenbogen group (36) has a >100‐fold selectivity for ERβ over ERα (36) and has demonstrated immunomodulatory, remyelinating, and neuroprotective effects in spinal cord and callosal axons (37). Ongoing studies by our lab demonstrate that IndCl ameliorates EAE clinical disease, motor symptoms, increases oligodendrocyte (OL) numbers, and promotes functional remyelination of callosal and spinal cord axons (38, 39, 40). In the current work, IndCl was tested for its therapeutic potential in the visual pathway. The novelty of this study lies in illuminating how an ERβ ligand can effectively mitigate disease within a specific CNS circuit once treatment starts at peak disease. Early longitudinal assessment of visual dysfunction along with early treatment prior to excessive axon damage might mitigate EAE visual deficits more effectively. Additionally, measuring visual function and recovery in the presence of novel therapies can be used to screen more effective therapies that will protect axons, stimulate axon remyelination, and prevent ongoing axon damage.

## 2 MATERIALS AND METHODS

### 2.1 Experimental animals

All procedures were conducted in accordance with the National Institutes of Health guidelines and approved by the Institutional Care and Use of Laboratory Animals Committee at the University of California, Riverside (UCR) and University of California, Los Angeles (UCLA). C57BL/6J wild‐type mice and Thy1‐YFP transgenic mice were purchased from the Jackson Laboratory. B6.Cg‐Tg(Thy1‐YFP)16Jrs/J mice (JAX #003709) were backcrossed to wild‐type C57BL/6 mice for more than five generations (41). PLP‐EGFP breeding pairs backcrossed to C57BL/6 mice, a kind gift provided by Dr. Wendy Macklin (University of Colorado, Denver, CO, USA), were bred and housed at UCLA and UCR vivarium facilities. The generation, characterization, and genotyping of these mice has previously been reported (42). Mice were all kept on a 12‐hour light/dark cycle with unrestricted access to food and water.

### 2.2 EAE induction and treatment

**FIGURE 1:**
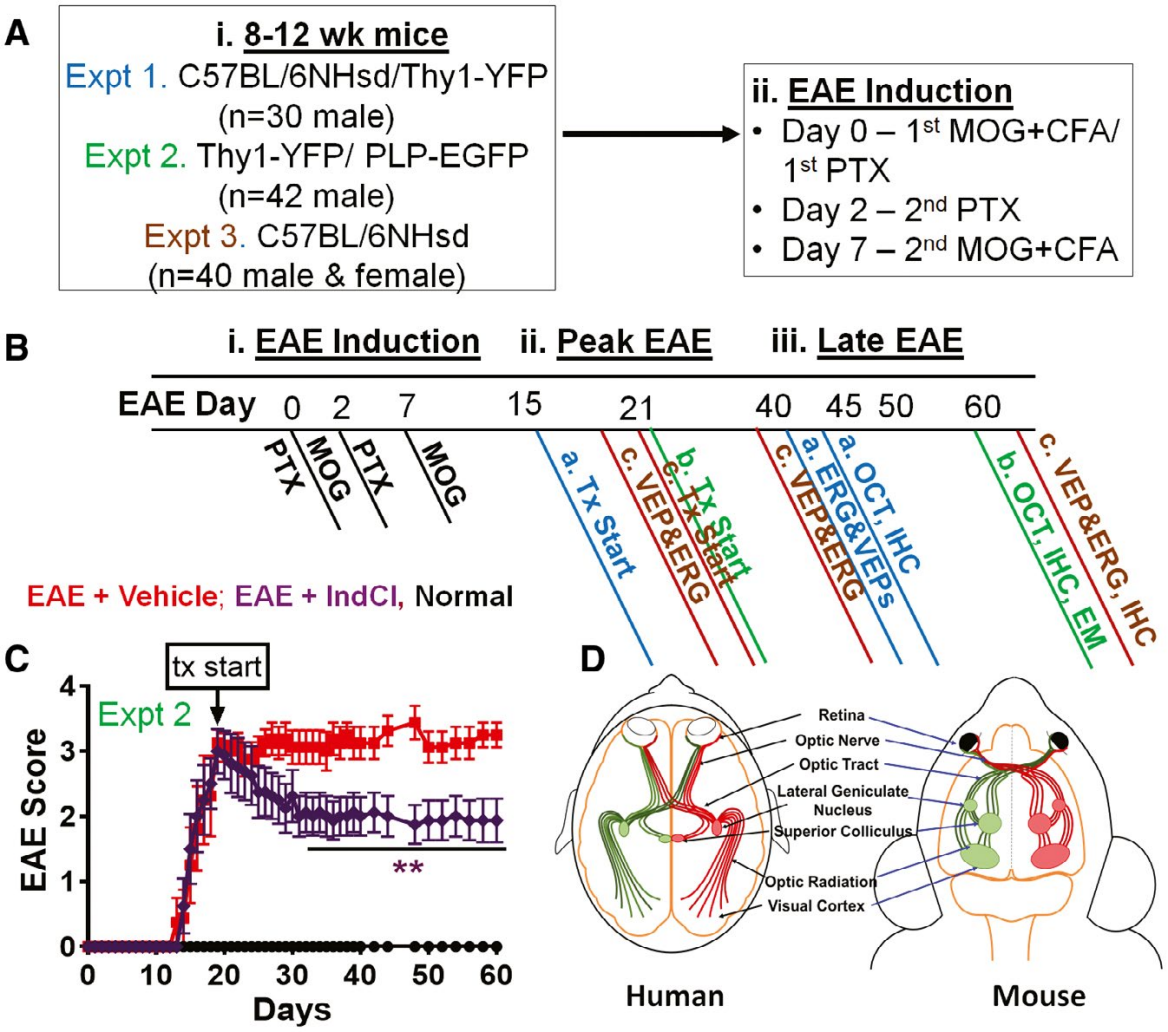
Schematic of MOG_35–55_ EAE. (A) Eight‐ to 12‐week‐old male and female C57BL/6NHsd/Thy1‐YFP (n = 10; total n = 30) mice in Experiment 1 (Expt 1; blue), Thy1‐YFP (n = 8/group; total n = 24), PLP‐EGFP (n = 6/group; total n = 18) mice in Experiment 2 (Expt 2; green), and C57BL/6NHsd (n = 10/group; total n = 40) mice in Experiment 3 (Expt 3; brown) were induced with EAE (i). EAE induction protocol involved initial MOG + CFA immunization and PTX injection on day 0, second PTX injection on day 2, and second MOG + CFA immunization on day 7 (ii). (B) After EAE induction mice received daily therapeutic treatment, beginning during peak EAE at 15 (Expt 1; blue), 18 (Expt 3; brown), 21 dpi (Expt 2; green) through the end of each experiment. Expt 1 and Expt 3 EAE groups received ERG and VEP visual function assessments and retinal imaging with OCT between day 40–45dpi (Expt 1 and 3) and day 60–65dpi (Expt 2 and 3). Tissue from Expts 1, 2, and 3 were processed for IHC and EM and only Expt 2 images are shown in the Figures 2, 3, 4, 5, 6. (C) Representative clinical scores for Thy1‐YFP and PLP‐EGFP age‐matched mice induced with EAE from Expt 2. Expts 1 and 3 followed similar EAE clinical disease. IndCl treatment started 21 dpi and was given daily at 5mg/kg via subcutaneous injection. Treatment groups included normal controls (black), EAE + Vehicle (10% ethanol, 90% miglyol; red), and EAE + IndCl (5mg/kg in vehicle; purple). Normal controls do not show any changes in clinical score from baseline. EAE + IndCl‐treated mice show decreased clinical scores over time, becoming significantly lower than EAE + Vehicle mice from 32 to 60 dpi. (D) A comparison of the anterior and posterior visual pathways between humans and mice is demonstrated since dysfunction in both pathways is a key aspect of MS pathology. Figure 1C data points and error bars represent mean + SEM. Experiment 1: n = 10–12 mice per group, Experiment 2: n = 10 mice per group, and Experiment 3: n = 8–12 mice per group with *p < 0.05, **p < 0.01. Statistical differences between groups were determined using two‐way unbalanced ANOVA with Dunnett’s multiple comparisons test

Eight‐ to 12‐week‐old male and female C57Bl/6J, Thy1‐YFP, and PLP‐EGFP mice were induced with EAE in three separate experiments and repeated twice as previously described and detailed in Supporting Information (28, 29) (Figure 1). Starting 7 days post induction (dpi), mice were scored daily for clinical disease severity. The clinical scoring protocol was defined as: 0, unaffected; 1, complete tail limpness; 2, failure to right upon attempt to roll over; 3, partial hind limb paralysis; 4, complete hind limb paralysis; and 5, moribund (28, 29).

Starting at peak disease 15–21 dpi, mice were scored and separated with even representation of clinical scores in the “vehicle” and “IndCl” treatment groups. IndCl (5 mg/kg body weight) was dissolved in vehicle (10% ethyl alcohol and 90% Miglyol 812N). Average mouse body weights were used to calculate the drug dosage. Vehicle and IndCl syringes and mouse cages were color coded for blinded treatment effects. Mice received daily drug or vehicle subcutaneous injections (Figure 1).

### 2.3 Optical coherence tomography

OCT data plots of mouse retinas from anesthetized animals were acquired with spectral domain‐OCT (R2200 840 nm HHP; Bioptigen) in tangent with Bioptigen InvivoVue software (Leica, Buffalo Grove, IL). Experimental details are presented in the Supporting Information. Retinal structure as assessed by OCT was analyzed just lateral to the optic nerve in order to minimize fluctuations in layer thickness observed near the optic nerve. Automatic segmentation of retinal layers was performed with Bioptigen Diver 3.0 software (Leica Microsystems). Software segmented individual retinal images avoided the inclusion of blood vessel diameter in RNFL calculations.

### 2.4 Electroretinograms and visual evoked potentials

Ocuscience Handheld Multi‐species ElectroRetinograph (Henderson, NV) was used to measure the changes in retinal and visual function in anesthetized dark adapted (5 hours) animals by recording ERGs and VEPs before midday (00:00–12:00). Experimental details are presented in the Supporting Information. ERG and VEP responses were averaged using Ocuscience ERG viewer. A minimum of 5 responses for ERGs and 25 responses for VEPs were averaged per mouse. The responses were then filtered for 60 Hz noise, along with a low‐pass filter of 150 Hz with MATLAB. Trace baselines were adjusted to 0 at the onset of the light stimulus. Only VEP responses were smoothed (3rd order Savitzky–Golay, 50 points per window) (43). MATLAB was used to identify and measure ERG and VEP peak amplitudes and latencies.

### 2.5 Perfusions, tissue preparation, and IHC

Mice were deeply anesthetized with isoflurane and intracardially perfused with ice‐cold PBS then 10% formalin in PBS (Fisher Scientific). Eyes, optic nerves, and brain were used for IHC as previously described and in the Supporting Information. Table S1 shows the antibodies used for IHC.

### 2.6 Microscopy, quantification, and statistics

Sections were imaged at similar light exposures using an Olympus BX61 spinning disk confocal microscope equipped with 10x and 40x Super Apochromat objectives (Olympus America Inc.) connected to a camera (Hamamatsu Orca‐R^2^). Z‐stack images were acquired, and projection images were compiled using Slidebook 6 and Cell sense software (Intelligent Imaging Innovations Inc). Immunofluorescence intensity and cell numbers were assessed with NIH ImageJ software (v1. 50i http://rsb.info.nih.gov/ij/) and quantified. Results from all counts were analyzed in GraphPad Prism for statistical significance.

### 2.7 Electron microscopy (EM)

Optic nerves were post‐fixed in 2% glutaraldehyde (Electron Microscopy Sciences) and 5% formalin for 24 hr (Fisher Scientific). They were processed for EM, imaged, and photographed as previously described (40). The number of myelinated and unmyelinated axons and the g‐ratio (the ratio of the axon diameter to the total myelinated fiber diameter), was quantified for at least 300 axons per group. For each axon, two measurements of axon diameters were made.

### 2.8 Statistics

All statistical analyses were performed using Prism 6.0 (Prism®, GraphPad) program for Windows and are described in detail in the Supporting Information.

## 3 RESULTS

### 3.1 IndCl treatment attenuates the development of retinal pathology

To evaluate the longitudinal EAE effects throughout the visual pathway, 8‐ to 12‐week‐old male and female C57Bl/6J, Thy1‐YFP, and PLP‐EGFP mice were induced with the MOG_35–55_ peptide EAE in three separate experiments, as previously described, with a subgroup not induced with EAE considered normal (Figure 1A) (28, 29, 37). Starting 7 dpi, EAE mice were scored daily for clinical disease severity. EAE disease onset occurred 10–13 dpi, reached a peak disease severity between 16 and 20 dpi, and maintained severity through late EAE, with euthanasia at 60 dpi (Figure 1B,C). Once mice reached peak disease, daily subcutaneous injections with an ERβ ligand, Chloroindazole (IndCl; 5 mg/kg; EAE + IndCl) or Vehicle (EAE + Vehicle) were performed. IndCl has been previously shown to reduce motor disability, and increase myelination and neuroprotection in the spinal cord therapeutically and prophylactically in EAE (39). However, its effects in the visual system have not yet been evaluated. Retinal structure and function were also evaluated at peak EAE and late EAE with OCT, VEP, and ERG recordings. Once IndCl treatment began, EAE + IndCl mice had decreased clinical disease severity compared to EAE + Vehicle mice (Figure 1C). A comparison of the anterior and posterior visual pathways between humans and mice has been demonstrated since dysfunction in both pathways has been a key aspect of MS pathology (Figure 1D).

**FIGURE 2:**
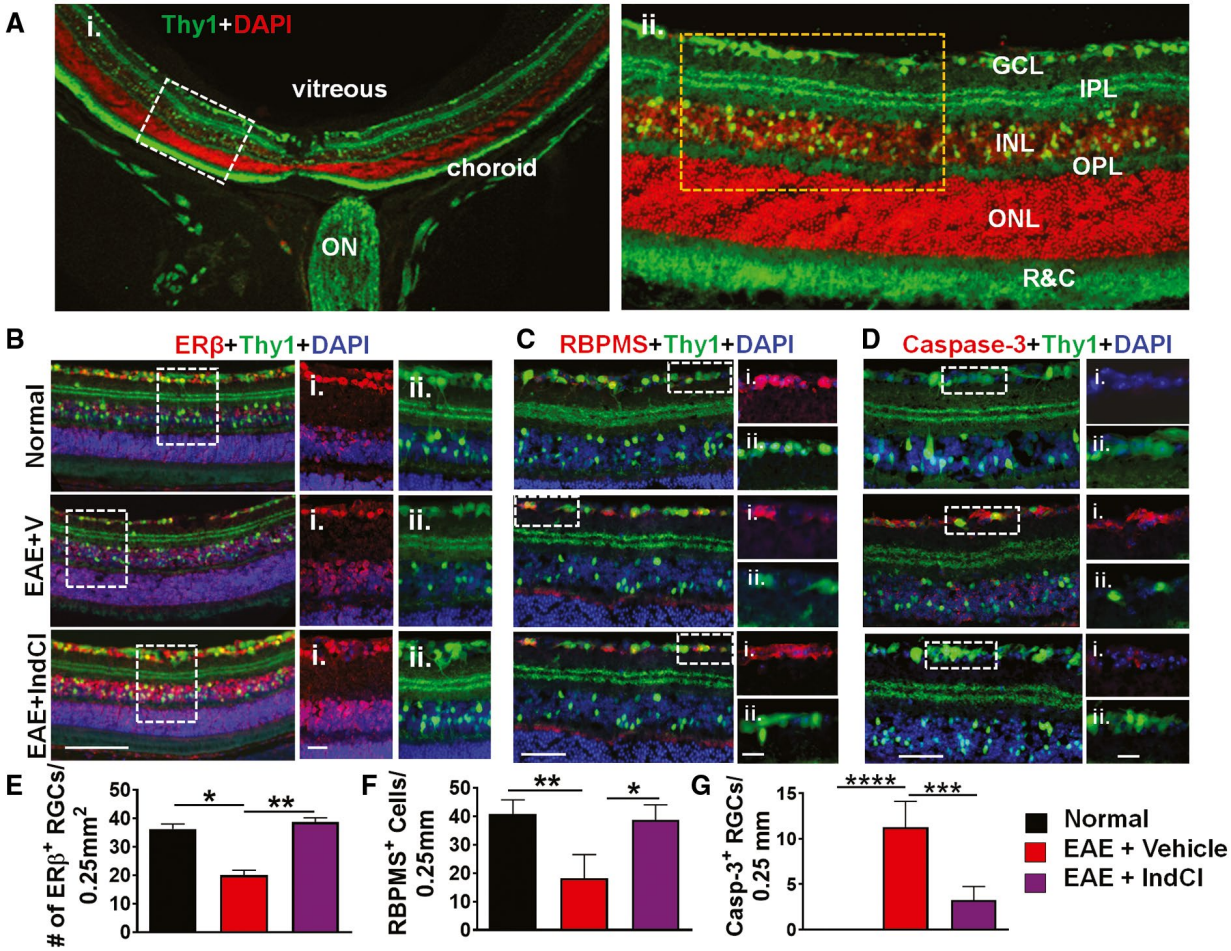
Therapeutic treatment with ERβ ligand IndCl improves RGC survival during EAE. (A) Representative 10x magnification image of retinas collected from normal Thy1‐YFP (green) mice and stained with DAPI (red). Vitreous, choroid, and optic nerve (ON) structures are identified for orientation. A magnified 20x image of the region indicated by the dashed rectangle in (A) located lateral to the optic nerve reveals individual retina layers including ganglion cell layer (GCL), inner plexiform layer (IPL), inner nuclear layer (INL), OPL, outer nuclear layer (ONL), and layer of rods and cones (R&C) (ii). (B) Thy1‐YFP (green) mouse retina sections collected 60 dpi from treatment groups shown in Figure 1C were immunostained for ERβ (red) and DAPI (blue). 20x magnified images show fewer Thy1^+^ neurons in the GCL of EAE + Vehicle, compared to normal and EAE + IndCl mice. (i; ii) Regions denoted by the dashed box were imaged at 40x magnification and show ERβ (i) and Thy1 (ii) for all groups. Thy1^+^ neurons in the GCL were also positive for ERβ. (C) Retina sections were immunostained with RNA‐binding protein with multiple splicing (RBPMS; red), and DAPI (blue). Sections were imaged at 40x magnification and analyzed in the region of interest indicated by the yellow dashed box in (A‐ii) to generate cell counts. Zoomed in split images showing RBPMS (i), and Thy1 (ii) for all treatment groups display a marked decrease in RBPMS^+^ RGCs in EAE + Vehicle mice compared to normal. EAE + IndCl mice show increased RBPMS^+^ RGCs compared to EAE + Vehicle mice. (D) Retina sections were immunostained with caspase‐3 (red). Split images show casp‐3 (i) and Thy1 (ii) for all groups. High levels of casp‐3 immunoreactivity in EAE + Vehicle mice are attenuated in EAE + IndCl mice. (E) Quantified cell counts show decreased numbers of ERβ^+^ GCL neurons in EAE + Vehicle mice compared to normal but an increase in these neurons in EAE + IndCl mice compared to EAE + Vehicle mice. (F) There was a decrease in RBPMS^+^ cells in EAE + Vehicle mice compared to normal, but an increase in RBPMS^+^ cells in EAE + IndCl mice compared to EAE + Vehicle mice. (G) There was an increase in apoptotic RGCs in EAE + Vehicle mice compared to normal mice, but this was alleviated in the EAE + IndCl mice. *n* = 8 mice per group. All graphs represent mean + *SEM*. *p < 0.05, **p < 0.01, ***p < 0.001, ****p < 0.0001 by ordinary one‐way ANOVA with Dunnett’s multiple comparison test. Scale bar is 100 µm for 20x image in (B), 50µm for (C) and (D), and 25µm for all zoomed in images.

EAE pathology was first assessed in the retina. The retina was isolated from normal, EAE + Vehicle, and EAE + IndCl groups of mice and sectioned along the horizontal plane. Thy1 immunoreactivity was evident in the GCL, IPL, INL, outer plexiform layer (OPL), and photoreceptor layer (Figure 2A). Horizontal midline retinal sections were used for immunostaining and imaged at 20X or 40X magnification lateral to the optic nerve (white dashed box in A) (Figure 2Aii). To assess the ERβ expression and to determine whether IndCl would be able to directly act on the retina, retinal sections from Thy1‐YFP normal, EAE + Vehicle, and EAE + IndCl groups were immunostained for ERβ. Robust ERβ expression was observed in RGCs of normal mice (Figure 2B). EAE + Vehicle mice demonstrated decreased ERβ immunoreactivity in the GCL compared to normal. However, EAE + IndCl mice showed increased ERβ immunoreactivity in all retinal nuclear layers compared to EAE + Vehicle. ERβ^+^ RGCs were quantified as a measure of ERβ^+^ Thy1^+^ neurons in the GCL. A decrease in the number of ERβ^+^ Thy1^+^ cells in the GCL of EAE + Vehicle mice was noted, compared to normal, and this decrease was attenuated with IndCl treatment (Figure 2E). RNA‐binding protein with multiple splicing (RBPMS), a selective marker for RGCs, was used to quantify RGCs (44), and robust immunostaining with RBPMS was observed in normal retinal sections (Figure 2C,F). EAE + Vehicle mice exhibited a decrease in RBPMS^+^ RGCs; however, this loss was mitigated with IndCl treatment. To determine if the decrease in ERβ^+^Thy1^+^ and RBPMS^+^ immunoreactivity in the GCL was due to EAE‐induced GCL cell death, immunostaining with the cell apoptosis marker, caspase‐3, was performed. Normal retinal sections revealed negligible caspase‐3 activity (Figure 2D); however, EAE + Vehicle mice showed a marked increase in EAE‐induced cell apoptosis in the RGC and INL, marked with the white dashed box, with this increase being reduced in EAE + IndCl retinal sections (Figure 2D,G). Retinal sections also demonstrated increased microgliosis (Iba‐1) and astrogliosis (GFAP) expression in the EAE + Vehicle mice, but these increases were also mitigated with IndCl treatment (Figure S1). Overall, EAE + IndCl mice demonstrated increased RGC survival compared to EAE + Vehicle mice.

### 3.2 IndCl‐treated EAE optic nerves show remyelination but persistence of axon damage

**FIGURE 3:**
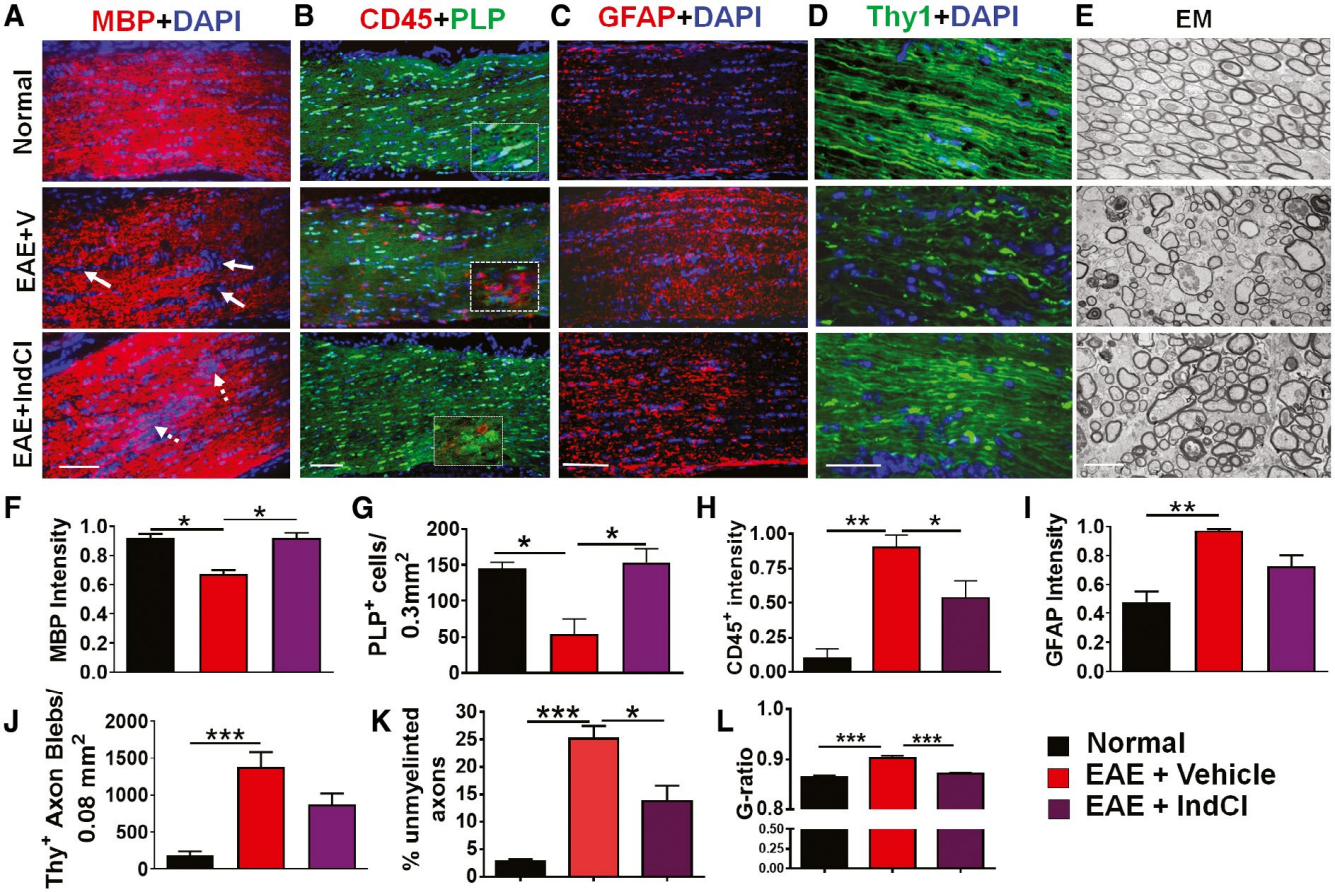
IndCl treatment attenuates demyelination, OL loss, and leukocytic infiltration, but not axonal degeneration in the optic nerve of EAE mice. (A‐E) Longitudinal optic nerve sections from normal, EAE + Vehicle, and EAE + IndCl mice at 60 dpi were immunostained for myelin basic protein (A; MBP; red), leukocytes with CD45 (B; red), and astrocytes with glial fibrillary acidic protein (C; GFAP; red) with nuclear stain DAPI (blue). Both PLP‐EGFP and Thy1‐YFP mice were used to assess OLs (B, proteolipid protein, PLP; green) and axons (D; green). (A) Coherent MBP immunostaining was disrupted and showed a significant decrease in EAE + Vehicle mice compared to normal. Treatment with IndCl significantly attenuated the decrease inMBP intensity as compared to EAE + Vehicle mice (A, F). The clusters of DAPI positive cells (identified by white arrows in A), which could be indicative of lesions in EAE + Vehicle mice, persisted even in the presence of IndCl treatment. Optic nerve sections from EAE + Vehicle mice showed an increase in CD45 immunostaining and a decrease in PLP‐EGFP OLs (B). IndCl treatment decreased CD45 intensity and increased PLP‐EGFP ^+^  OLs (B, G, H). GFAP immunoreactivity from normal mice reveal small astrocytes aligned along the horizontal axis (C). EAE + Vehicle mice show an increase in GFAP reactivity with disruptions in the linear organization of astrocytes. IndCl treatment did not have a significant effect on GFAP immunoreactivity compared to EAE + Vehicle (C, I). (D, J) Thy1‐YFP (green) optic nerve sections imaged at 40x magnification show an increase in Thy1‐YFP^+^  axons with blebbing, swelling, and transections in EAE + Vehicle mice as compared to normal. IndCl treatment did not attenuate the axon blebbing and swollen axons although more intact axons remained. (E) Optic nerves from normal, EAE + Vehicle and EAE + IndCl treatment groups were collected at 60 dpi and prepped for EM to assess individual optic nerve axons. Cross‐sectioned optic nerve micrographs reveal coherent myelinated axons in the normal mice. EAE + Vehicle mice show significant axon damage and demyelination. EAE + IndCl mice shows increased numbers of myelinated axons and overall improvement of axon morphology compared to EAE + Vehicle. However, the presence of significant damaged axons persisted. (K) Quantification of the number of unmyelinated axons reveals an eightfold increase in EAE + Vehicle compared to normal. Optic nerves from EAE + IndCl mice show an approximately 45% decrease in the number of unmyelinated axons compared to EAE + Vehicle. (L) Calculated g‐ratios are significantly increased in the EAE + Vehicle mice compared to normal mice. This effect is attenuated with the EAE + IndCl mice, representative of increased axon myelination in the optic nerve. All graphs represent mean + SEM. n = 4–6 mice per group. *p < 0.05, **p < 0.01, ***p < 0.001 with ordinary one‐way ANOVA using Kruskal–Wallis multiple comparison test. Scale bar is 100µm for A–D and 2.5µm for E

To assess the changes in demyelination, inflammation, and neurodegeneration in the optic nerve, longitudinal optic nerve sections from normal PLP‐EGFP and Thy1‐YFP controls, EAE + Vehicle, and EAE + IndCl mice were evaluated using IHC (Figure 3). Myelin Basic Protein (MBP) immunoreactivity was used to evaluate axon myelination in longitudinal optic nerve sections. Sections from normal mice showed robust MBP staining with a relatively consistent distribution of DAPI^+^ nuclei (Figure 3A). In contrast, significant decreases in myelin intensity as well as lesions containing increased numbers of DAPI^+^ cells were observed in EAE + Vehicle mice, as indicated by white arrows (Figure 3A,F). EAE + IndCl mice showed enhanced MBP immunoreactivity with fewer demyelinated lesions compared to EAE + Vehicle mice; however, there remained a persistent increase in DAPI^+^ nuclei indicated by the dashed white arrows (Figure 3A,F).

To assess mature OLs, leukocytes, and astrocytes, PLP‐EGFP optic nerve sections were immunostained for CD45, a pan leukocyte marker, and GFAP (Figure 3B,C). Normal mice showed a robust population of PLP^+^ OLs compared to EAE + Vehicle mice, where a significant reduction of PLP^+^ OLs was observed. EAE + IndCl mice showed a significant recovery in the number of PLP‐EGFP OLs as compared to EAE + Vehicle animals (Figure 3B,G). Normal optic nerve sections showed a negligible number of CD45^+^ leukocytes and a baseline level of GFAP^+^ astrocytes, whereas EAE + Vehicle mice exhibited extensive CD45^+^ leukocyte immunoreactivity and astrogliosis (Figure 3B,C,H,I). Sections from EAE + IndCl mice showed a significant decrease in CD45^+^ leukocytic immunoreactivity but not astrogliosis, and an increase in PLP^+^ cells compared to EAE + Vehicle (Figure 3B,C,G,H,I).

The onset of demyelination in the optic nerve is associated with axon damage (45). Optic nerve longitudinal sections from Thy1‐YFP mice were imaged to assess optic nerve axon health. Optic nerve sections from normal mice showed robust Thy1‐YFP fluorescence, whereas EAE + Vehicle and EAE + IndCl Thy1‐YFP optic nerves revealed a decrease in Thy1‐YFP fluorescence, with increased numbers of punctate, fragmented “axonal blebs” (Figure 3D,J).

To further assess axon myelination integrity, ultrastructure EM analysis of optic nerve cross‐sections from different groups was performed. All myelinated and nonmyelinated axons within a given field were used to assess the mean ratio of inner axonal diameter to total outer diameter (g‐ratio) (Figure 3E). Normal optic nerve sections had mostly myelinated axons with a mean g‐ratio of 0.86 ± 0.12 and 3.00 ± 0.78% of nonmyelinated axons. Vehicle‐treated EAE optic nerve sections revealed an increased number of demyelinated axons (25 ± 4.6%) and a higher g‐ratio (0.90 ± 0.12). Despite the presence of intact myelin in some axons, a majority of axons showed signs of degeneration. Many axons appeared shrunken and distorted, with dense granules and with thin or un‐compacted myelin; some myelin bundles lacked the presence of axons altogether. IndCl treatment during peak disease showed a significant decrease in both the g‐ratio (0.87 ± 0.09) and the percent of nonmyelinated axons (13 ± 6%) (Figure 3K,L). However, many of the axon and myelin pathologies persisted, similar to those seen in EAE + Vehicle sections.

### 3.3 IndCl treatment increases myelination but partially alleviates axon damage in the optic tract

**FIGURE 4:**
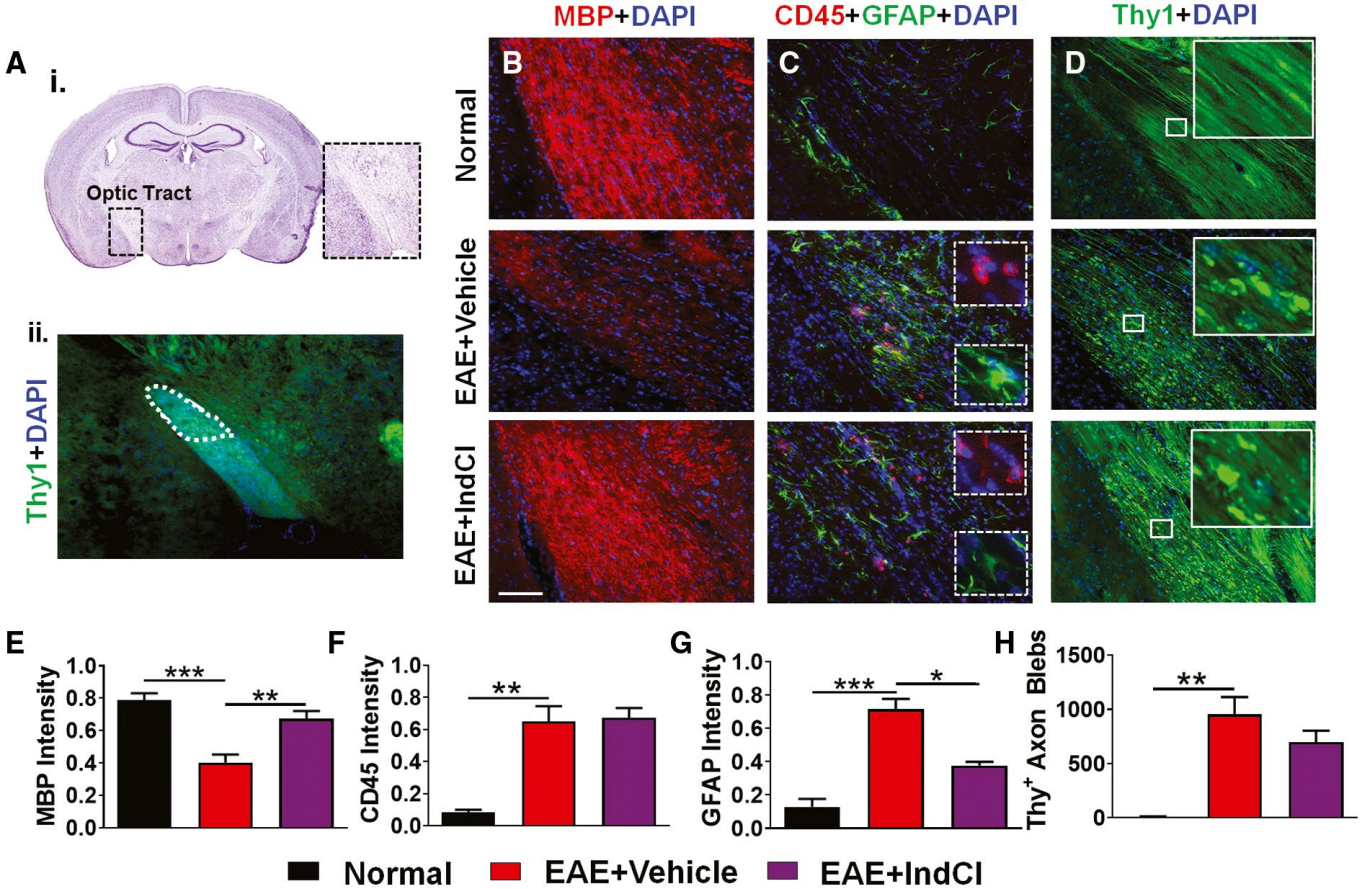
IndCl treatment ameliorates myelin loss and astrogliosis but not leukocytic infiltration in the optic tract of EAE mice. (A) Nissl‐stained mouse coronal sections that have a distinct optic tract from plate 43 of the Paxinos and Franklin atlas (47) (i). The area encompassed by the black dashed box highlights the location of the optic tract. (ii) 10x magnified image of the optic tract region in normal male Thy1‐YFP mouse, with the optic tract traced to highlight region of analysis. (B) Coronal sections containing optic tracts from normal, EAE + Vehicle, and EAE + IndCl male Thy1‐YFP mice were collected 60 dpi. (B) Sections were immunostained for MBP (red) and imaged at 20x magnification. (E) MBP immunofluorescence is decreased in EAE + Vehicle mice, an effect mitigated with IndCl treatment. (C) Sections immunostained for CD45 (red), GFAP (green), and DAPI (blue) were imaged at 20x magnification. EAE + Vehicle mice show increased CD45 and GFAP immunoreactivity compared to normal. EAE + IndCl mice show decreased GFAP but not CD45 immunofluorescence. White dashed rectangle insets show magnified CD45^+^ cells (top) and GFAP^+^ astrocytes (bottom) in EAE + Vehicle and EAE + IndCl mice. (D) Thy1‐YFP sections were assessed for axonal integrity. Normal tissue shows coherent healthy axons, however axon damage indicated by axon fragmentation and swelling is evident in EAE + Vehicle mice. Sections from EAE + IndCl mice show a combination of fragmented swollen axons and intact axons. White rectangle insets show magnified images of Thy1^+^ intact axons in normal, fragmented, and swollen axons in EAE + Vehicle, and a combination of fragmented and intact axons in EAE + IndCl mice. (E) MBP staining intensity is decreased in the vehicle group compared to normal but increased in the IndCl group compared to vehicle. (F) CD45 intensity is increased in the EAE + Vehicle group compared to normal, and not changed between EAE + Vehicle and EAE + IndCl mice. (G) GFAP staining intensity is significantly increased in EAE + Vehicle compared to normal, however the EAE + IndCl mice show decreased staining intensity compared to EAE + Vehicle. (H) The number of Thy1^+^ axon blebs is minimal in normal, significantly increased in vehicle but not attenuated in IndCl treatment groups. n = 4–6 mice per group. All graphs represent mean + *SEM*. *p < 0.05, **p < 0.01, ***p < 0.001 with ordinary one‐way ANOVA using Kruskal–Wallis multiple comparison test. Scale bar is 100 µm

In the optic tract, RGC axons remain heavily myelinated and are susceptible to EAE‐induced inflammation, demyelination, and axon damage. Effects of EAE‐induced inflammation and astrocytic activation in the optic tract were investigated in brain sections corresponding to plates 43–45 of the Paxinos and Franklin brain atlas and imaged in the dorsal region of the optic tract (Figure 4A‐i,‐ii). Normal, EAE + Vehicle, and EAE + IndCl brain sections were immunostained with MBP to assess axon myelination. A significant decrease in MBP immunoreactivity was observed in the optic tract of EAE + Vehicle mice as compared to normal optic tract sections, and this decrease was attenuated in EAE + IndCl mice (Figure 4B,E). Optic tracts were also immunostained for CD45 and GFAP (Figure 4C,F,G). Both staining intensities were significantly increased in EAE + Vehicle mice compared to normal. While EAE + IndCl mice did not demonstrate modified CD45 immunostaining, IndCl treatment did decrease GFAP immunostaining intensity in the optic tracts.

Thy1‐YFP fluorescence was imaged in the dorsal region of the optic tract to determine if RGC axonal degradation observed in optic nerve sections extended to the optic tract. Axon fragmentation and blebbing were ubiquitous in optic tract sections from EAE + Vehicle mice as compared to coherent and intact Thy1^+^ axons in normal, but treatment with IndCl did not attenuate EAE‐induced axon pathology (Figure 4D). Quantification of the punctate “axon blebs” showed significant increases in EAE + Vehicle mice as compared to normal, with no difference in the EAE + IndCl mice (Figure 4H).

### 3.4 Demyelination and inflammation persist in IndCl‐treated EAE dorsal LGN

**FIGURE 5:**
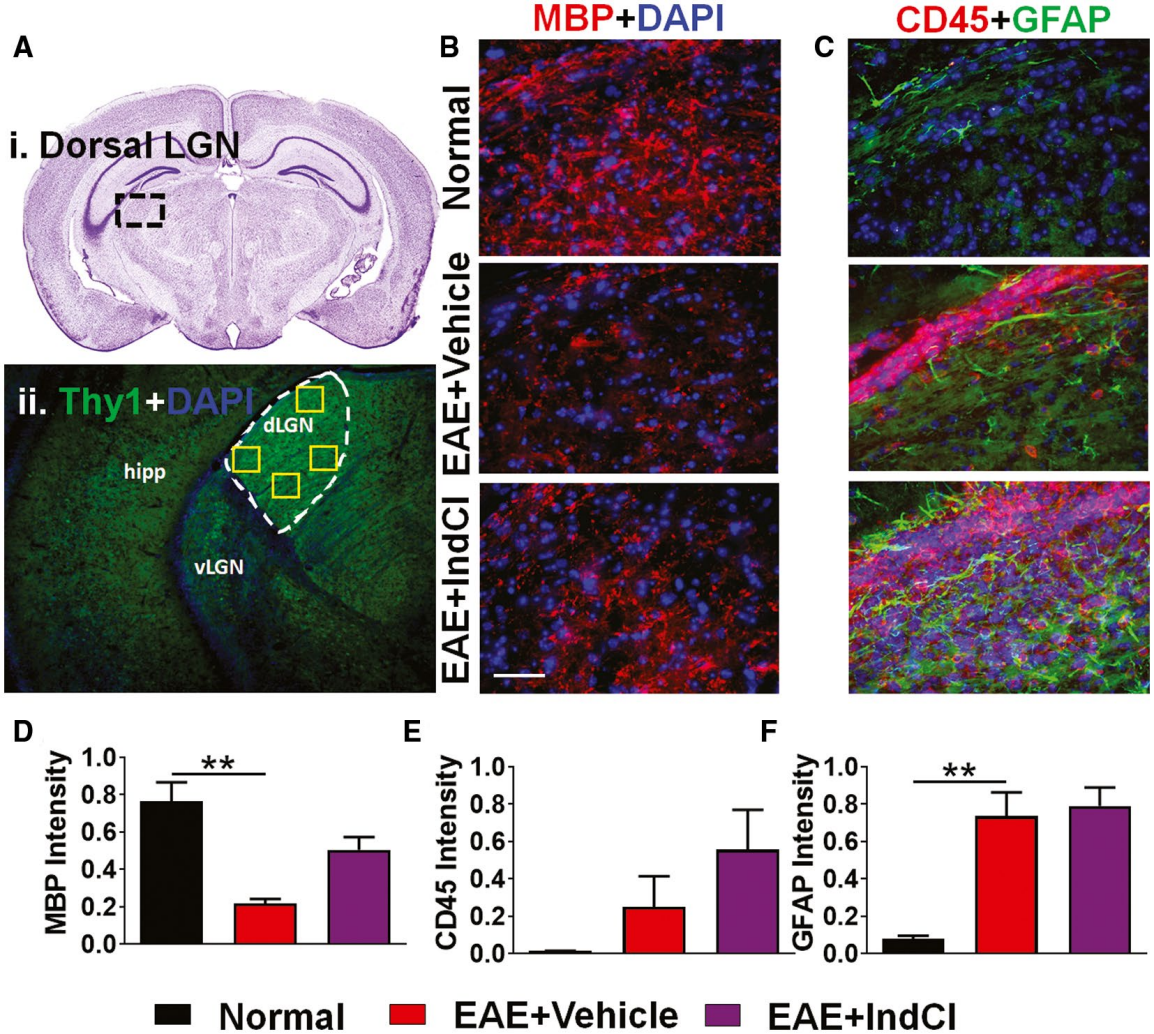
IndCl treatment does not improve myelin loss or astrogliosis in the dLGN. (A) Nissl‐stained mouse coronal sections containing the dLGN from plate 51 of the Paxinos and Franklin atlas (i). The black dashed box highlights the location of the dLGN within the larger section. (ii) The dLGN is traced to highlight the region of analysis immunostained with MBP (red) and DAPI (blue), in (B). (B, D) Coronal sections containing the dLGN were immunostained with MBP (red) to assess myelination. Decreased dLGN MBP immunoreactivity was observed in EAE + Vehicle mice as compared to normal mice. Treatment with IndCl did not modify the loss of MBP seen in the EAE + Vehicle mice. (C, E, F) dLGNs immunostained with CD45 (red) and GFAP (green) show an increase in CD45^+^ cells in EAE + Vehicle mice that did not reach significance. However, GFAP immunoreactivity increased significantly in EAE + Vehicle mice as compared to normal mice. n = 4–5 mice per group. All graphs represent mean + *SEM*. **p < 0.01 with ordinary one‐way ANOVA with Kruskal–Wallis multiple comparison test

RGC axons from the retina project to a number of synaptic targets in the visual system, one of which is the LGN. The dorsal LGN (dLGN) is an important processing hub and relay station that sends and receives geniculocortical projections to the visual cortex where higher order visual processing occurs (46). In order to evaluate the effect of EAE on the dLGN, coronal sections were obtained from Thy1‐YFP mice, corresponding to plates 51–53 of the Paxinos and Franklin mouse brain atlas (Figure 5A‐i) (47). Sections were immunostained, then imaged at 40x magnification in four locations within the dLGN indicated by the yellow squares (Figure 5A‐ii). Results of immunohistochemical analysis in each of the four regions were averaged for quantification. For comparison, a key locus in the efferent pathway, ventral LGN that is not part of the afferent pathway was also analyzed (Figure S2).

MBP immunostaining revealed sparse myelination in the dLGN (Figure 5B). A significant decrease in MBP immunoreactivity was observed in the dLGN of EAE + Vehicle mice as compared to normal, and IndCl treatment did not alleviate this MBP staining deficit (Figure 5B,D). Leukocyte assessment with CD45 immunoreactivity showed a small increase in reactivity in EAE + Vehicle mice, and an increase compared to vehicle in EAE + IndCl mice, neither of which reached significance (Figure 5C,E). However, GFAP immunoreactivity revealed increased astrocyte activation in EAE + Vehicle mice as compared to normal (Figure 5C,F). IndCl treatment did not change the number of astrocytes, which remained similar to the EAE + Vehicle mice.

### 3.5 Mitigation of EAE‐induced visual cortex demyelination in the presence of IndCl

**FIGURE 6:**
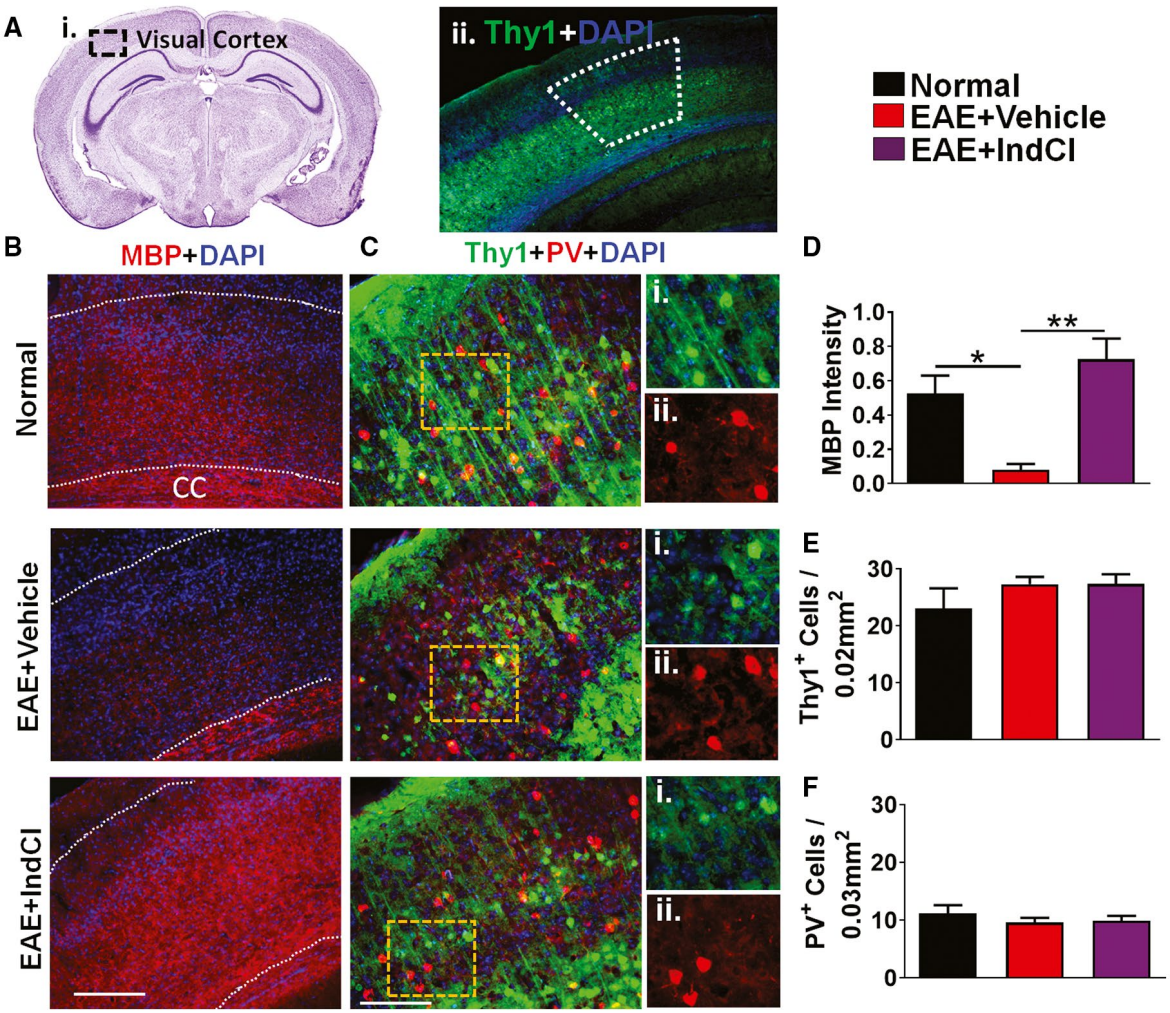
Treatment with IndCl attenuates myelin loss in the visual cortex of EAE mice. (A) Nissl‐stained mouse coronal sections containing the visual cortex are evident in plate 51 from the Paxinos and Franklin atlas (i). The area encompassed by the black dashed box highlights the location of the visual cortex within the larger section. (ii). (B) Brain sections immunostained with MBP (red) were imaged at 10x magnification and analyzed in the region of interest corresponding to primary visual cortex layers 2–6. (D) A decrease in MBP immunoreactivity was observed in EAE + Vehicle mice compared to normal. Treatment with IndCl attenuated the MBP decrease observed in EAE + Vehicle mice. (C) Thy1^+^ (green) coronal sections containing the visual cortex were immunostained for parvalbumin (PV; red) and DAPI (blue) and imaged at 20x magnification. Magnified split images show Thy1 (i) and PV (ii) for all treatment groups. (E, F) There were no difference in Thy1^+^ or PV^+^ cell bodies between groups. However, Thy1^+^ axonal projections in EAE + Vehicle mice show less processes as compared to normal. Treatment with IndCl caused a smaller loss of projections as compared to EAE + Vehicle mice. n = 4–6 mice per group. All graphs represent mean + *SEM*. *p < 0.05, **p < 0.001 using ordinary one‐way ANOVA with Kruskal–Wallis multiple comparison test. Scale bar is 200 µm

Thalamocortical neurons in the LGN project to layer 4 and synapse on resident spiny stellate neurons of the visual cortex. In mice and humans, the visual cortex is a major processing center for thalamocortical inputs and is highly myelinated. Here, axon myelination and neuronal changes were assessed in the visual cortex of Thy1‐YFP coronal sections corresponding to plates 52–54 of the Paxinos and Franklin mouse brain atlas (Figure 6A‐i) (47). Sections were analyzed in the demarcated region of interest, which included layers 2–6 (Figure 6A‐ii). EAE + Vehicle mice showed a decrease in MBP immunoreactivity compared to normal (Figure 6B,D), and this decrease was fully reversed in the EAE + IndCl mice (Figure 6B,D). Since Thy1 is expressed in a subset of cortical pyramidal neurons (48), the next step was to determine neuronal counts in the visual cortex. Both layers 5 and layer 2/3 pyramidal neurons in the visual cortex expressed the fluorescent protein, but only layer 2/3 was quantified due to the high density of layer V neurons. Visual cortex‐containing sections from the normal group showed significant numbers of Thy1^+^ pyramidal neurons with intact apical dendrites (Figure 6C). EAE + Vehicle mice showed no changes in the number of Thy1^+^ cell bodies (Figure 6E); however, fewer apical dendrites were evident (Figure 6C). EAE + IndCl mice also did not show any difference in the number of Thy1^+^ cell bodies compared to EAE + Vehicle (Figure 6E), but improvements in Thy1^+^ apical dendrite density were observed (Figure 6C). Parvalbumin (PV)‐positive interneurons regulate synchrony among neuronal networks and are important for maintaining spike timing in the visual cortex (49). PV interneuron inactivation results in decreased synchrony of neuronal networks. No differences in number of PV^+^ neurons were observed between normal, EAE + Vehicle, and EAE + IndCl mice (Figure 6C,F).

### 3.6 Alleviation of EAE‐induced functional deficits with therapeutic treatment with IndCl

**FIGURE 7:**
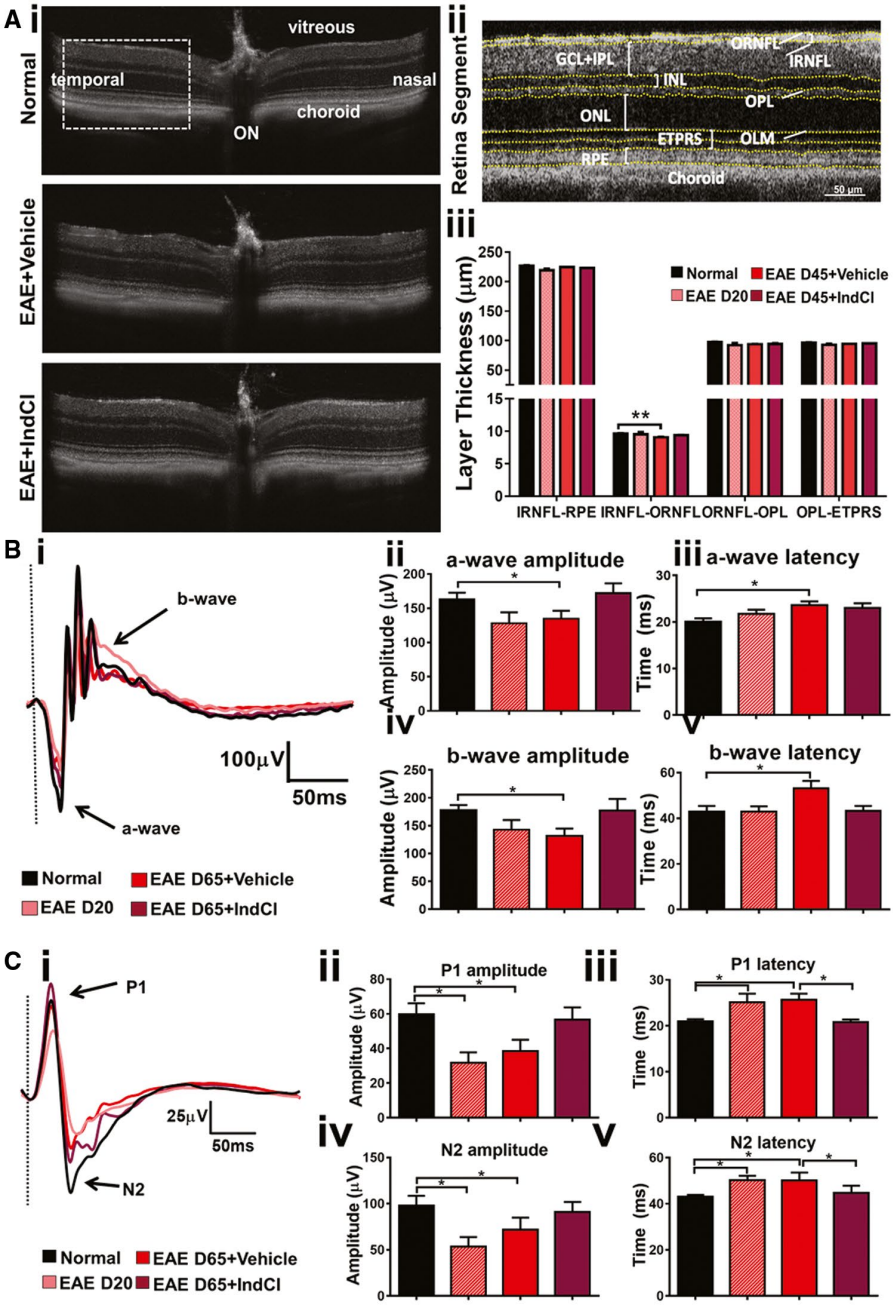
IndCl treatment does not change retinal layer size with OCT or ERG latency, but decreases VEP latency. (A) Representative C57Bl/6 mice OCT retina scans of normal, EAE D45 + vehicle, and EAE D45 + IndCl (Expt 1), with optic nerve, choroid, vitreous, temporal, and nasal regions demarcated in the normal image. OCT scans show no obvious differences in retina morphology between treatment groups. (ii) The region indicated by the dashed rectangular box in (A), just lateral to the optic nerve, was segmented into individual layers to detect small changes in individual layer thicknesses that were not readily observable by eye. Automatic segmentation software demarcated and calculated individual layer thicknesses of the whole retina, RNFL, IPL, INL, OPL, outer nuclear layer (ONL), the outer limiting membrane (OLM), the photoreceptor end tips (ETPRS) retinal pigment epithelium (RPE), and choroid. (iii) Quantification of retina layer thicknesses shows no differences in whole retina (RNFL‐RPE), combined ORNFL‐OPL, or combined OPL + ETPRS layers between groups. EAE D45 + vehicle mice show decreased RNFL thickness compared to normal. IndCl‐treated mice showed no significant difference in RNFL, or other layer thickness compared to EAE D45 + vehicle. (B) Retina function was assessed by recording ERGs from normal, EAE + Vehicle, and EAE + IndCl mice (Expt. 3). Recording silver thread electrodes were placed on the cornea, reference needle electrodes in the snout, and a ground needle electrode in the tail. (B‐i) Sample ERG responses from treatment groups are shown with a‐wave and b‐wave demarcated by the black arrows. No significant differences in a‐wave amplitude, a‐wave latency, b‐wave amplitude, and b‐wave latency were observed between EAE Day 20 and normal mice (B‐ii, iii, iv, v). However, EAE + Vehicle at 65dpi mice exhibited a decrease in a‐wave and b‐wave amplitude and an increase in a‐wave and b‐wave latency compared to normal, that was not alleviated with IndCl treatment. (C) Cortical visual function was assessed by recording VEPs from normal, EAE + Vehicle, and EAE + IndCl mice (Expt. 3). Recording needle electrodes were placed subdermally above the visual cortex, reference needle electrodes in the snout, and a ground needle electrode in the tail. (C‐i) Sample VEP responses from all treatment groups are shown with P1 and N2 components demarcated by the black arrows. (C‐ii, iv) EAE 20 dpi mice exhibited a decrease in P1 and N2 amplitudes compared to normal. These decreases were maintained in the EAE + Vehicle mice 65 dpi. The IndCl‐treated group did not show a significant effect on either P1 or N2 amplitudes. (C‐iii, v) However, there was an increase in P1 and N2 latencies in EAE 20 dpi and EAE 65 dpi groups compared to normal. The IndCl‐treated EAE group showed a significant decrease in both P1 and N2 latencies as compared to EAE + Vehicle mice. (A): *n* = 8‐10 mice per group. (B): n = 8–10 per group. (**C**): n = 12 mice per group. All graphs represent mean + *SEM*. *p < 0.05 using ordinary one‐way ANOVA with Kruskal–Wallis multiple comparison test

EAE was induced in a separate group of C57BL/6NHsd mice to assess changes in retinal structure and function by OCT, ERG, and VEP (Figure 1A,B). IndCl treatment was initiated at 20 dpi, at the peak of clinical disease, and mice were subjected to OCT imaging at 45 dpi. The temporal retina was imaged approximately 0.3 mm lateral to the optic nerve (Figure 7A‐i). Automatic segmentation of OCT images by system software was used to demarcate the different layers (RNFL, GCL, IPL, INL, OPL, ONL, ETPRS, and RPE) of the retina and measure their respective thicknesses in an unbiased and accurate manner (Figure 7A‐ii). The GCL and IPL were combined for analysis due to the difficulty associated with differentiating the two layers using OCT. Similar to the OCT analysis for human patients, retina interneuron layers (IPL + INL) and photoreceptor layers (ONL + ETPRS) were grouped for simplicity (15).

Whole retina (RNFL to RPE), GCL + IPL, ORNFL to OPL, and OPL to ETPRS measurements did not show significant differences between normal and EAE + Vehicle mice (Figure 7A‐iii). EAE + Vehicle mice showed a significant decrease in the RNFL thickness as compared to normal at 45 dpi with no changes in the IndCl group.

As demonstrated in the preceding histological analysis of afferent visual system nuclei and tracts, significant demyelination, inflammation, and axonal damage are present in the visual pathway during EAE, some of which IndCl treatment attenuates. However, whether these effects result in functional changes in visual performance required further assessment. ERG and VEPs were recorded at peak disease (20 dpi) before treatment was started (EAE D20), and then, at 65 dpi after treatment with either IndCl (EAE D65 + IndCl) or vehicle (EAE D65 + vehicle).

The ERG provides an assessment of retinal function and has been well characterized. The initial downward deflection of the voltage is identified as the a‐wave and originates within the photoreceptors, while the fast upward deflection, identified as the b‐wave, is generated by some retinal interneuron subtypes, Müller glia, and RGC firing (50). When mice are dark‐adapted, ERG and VEP responses are primarily driven by rod pathways (51).

To characterize the functional consequence of retinal pathology during EAE, retinal function in mice was assessed by recording ERGs in live, anesthetized, and dark‐adapted C57BL/6 mice. ERG responses of normal mice show a characteristic downward deflection of the a‐wave of about 200 µV in amplitude, then an immediate ~500 µV increase in amplitude characteristic of the b‐wave, followed by a gradual return to baseline (Figure 7B‐i). EAE D65 + Vehicle mice had significantly decreased ERG a‐wave and b‐wave amplitudes, and increased a‐wave and b‐wave latencies compared to normal. EAE D65 + IndCl mice showed no significant differences in ERG parameters compared to EAE + Vehicle mice (Figure 7B‐ii‐v).

As a whole, VEP measurements are accepted to be an assessment of afferent visual function. Thus, dysfunction in any component of the visual pathway affects the VEP response (51). Mouse VEP traces have an initial upward deflection referred to as P1, followed by a sharp downward deflection referred to as N2, followed by a return to baseline. Amplitude changes of peak components are primarily caused by axonal damage and neuronal dysfunction (52), whereas latency changes are thought to arise from primarily demyelinating events (53).

The functional effects of visual pathway pathology were measured by recording VEPs using subdermal electrodes resting on the skull above the visual cortex. In normal mice, VEPs show over 50 µV positive deflection, then a rapid 50 µV decrease in voltage, followed by a return to baseline (Figure 7C‐i). EAE D20 + Vehicle and EAE D65 + Vehicle mice showed a significant 50% decrease in P1 and N2 amplitudes compared to normal, as well as a significant increase in latency (Figure 7C‐ii‐v). EAE D65 + IndCl mice demonstrated no significant changes in P1 and N2 amplitude and N2 latency as compared to EAE D65 + Vehicle mice. However, P1 and N2 latency was significantly decreased in EAE D65 + IndCl mice as compared to EAE D65 + Vehicle mice (Figure 7C‐iii,v).

## 4 DISCUSSION

**FIGURE 8:**
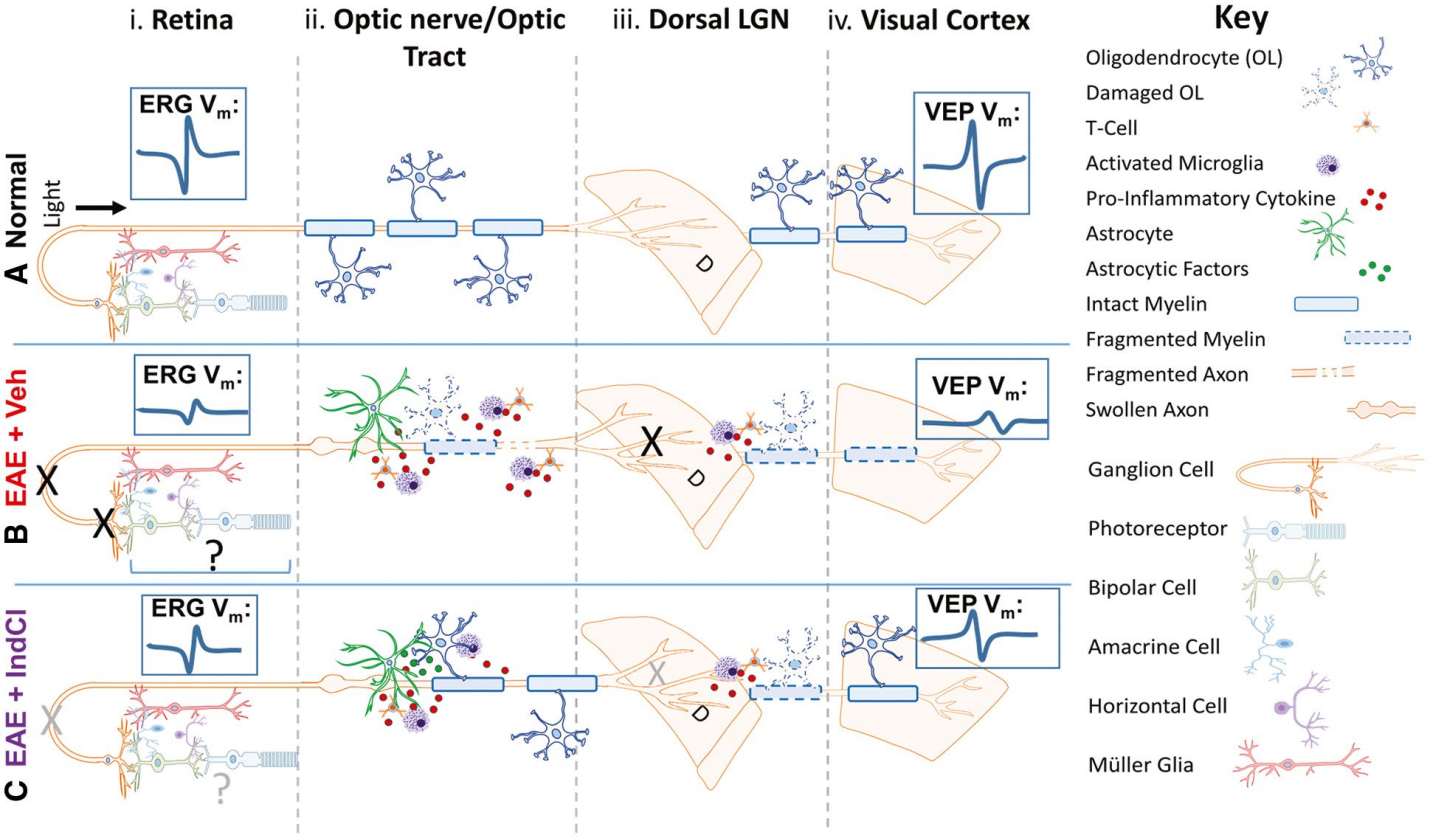
Model of visual pathway dysfunction and IndCl effects on the visual system during EAE. (A) Schematic depicting normal visual pathway. (i) Photoreceptors form synapses with bipolar cells which then synapse onto RGCs. Photoreceptor/bipolar cell and bipolar cell/RGC synaptic transmission is modulated by amacrine and horizontal cells. Müller glia span the neural portion of the retina and interact with the neural retina. ERGs amplitudes are robust with normal parameters. (ii) Mature OLs myelinate most RGC axons in the optic nerve and optic tract thereby providing trophic support and improving axon conduction. (iii) RGC axon terminals form synapses with thalamocortical neurons in the dLGN. Myelinated thalamocortical neurons in the dLGN send projections to the primary visual cortex. (iv) With no disruption of the afferent visual system, VEP amplitudes are robust with normal parameters. (B) Schematic depicting EAE visual pathway. (i) In EAE, RGC cell bodies and their axons comprising the retinal nerve fiber layer are damaged. ERG amplitudes are decreased, but response latencies remain similar to normal. EAE‐induced effects on photoreceptors, retinal interneurons, and Müller glia effects require further study. (ii) Breakdown of the blood–brain barrier during EAE results in leukocytic infiltration into the optic nerve and optic tract. The inflammatory milieu including pro‐inflammatory factors from lymphocytes macrophages, and other immune cells leads to astrocytic activation, myelin loss, axonal swelling and axonal degradation. (iii) Axonal degradation in anterior regions of the visual pathway likely lead to a loss of synaptic input in the dLGN. Leukocytic infiltration into the dLGN leads to a loss of myelination. Thalamocortical projection neurons of the dLGN are likely demyelinated decreasing signal transmission to the visual cortex. (iv) Significant defects in the anterior portion of the visual pathway, and a loss of myelination in the visual cortex lead to decreased VEP amplitudes and slight increases in response latencies. (C) Schematic depicting proposed effects of IndCl on the EAE visual pathway. (i) IndCl treatment protects RGC somas but does not affect intraretinal RGC axons comprising the RNFL. ERG latencies and not amplitudes are improved with IndCl treatment. IndCl effects on photoreceptors, retinal interneurons, and Müller glia need further investigation. (ii) IndCl treatment results in partial decreases in leukocytic presence in the optic nerve, and astrocytic activation in the optic tract. IndCl promotes myelination in the optic nerve and optic tract. This effect is not sufficient to prevent RGC axon degradation. (iii) Smaller but significant axonal damage in anterior visual pathway, even with robust remyelination, likely leads to a loss of synaptic input to the dLGN. Minimal remyelination in the dLGN indicates that EAE‐induced deficits to thalamocortical projections are likely not improved by IndCl treatment. (iv) Although the visual cortex and anterior regions of the visual pathway show improved myelination with IndCl treatment, this results in improved VEP latencies but not amplitudes.

In the present study, MOG‐EAE was used to investigate MS‐related visual dysfunction. It is the first study to assess EAE‐induced pathology in all major nuclei and tracts of the afferent visual pathway as well as the therapeutic potential of the ERβ ligand IndCl for attenuating these effects. Our results indicate that chronic EAE caused functional deficits throughout the visual pathway (assessed with ERG and VEP) in addition to inflammation, demyelination, and axon damage. IndCl treatment increased axon myelination and mitigated axon damage, which led to some functional improvement measured with ERG and VEP (summarized in Figure 8).

Approximately 50% of patients suffering from MS experience ON prior to exhibiting initial symptoms, while 70% develop the disorder at some point during disease progression (12). MS patients that do not report incidences of ON often still have some form of visual dysfunction such as impaired contrast sensitivity, low‐contrast letter acuity, and impaired color discrimination (12, 54, 55). These visual deficits in patients have been associated with RNFL thinning, loss of RGCs, and increased latency in VEPs (13). Although the human and mouse visual systems differ in complexity, they share core similarities (Figure 1D, modified from (56)). Table 1 shows some similarities and differences in findings from MS postmortem tissue and EAE within the visual pathway.

**TABLE 1:** Comparing the visual system results in EAE and MS

	MS	MOG_35–55_ peptide EAE
Retina	RNFL thinning (13, 15, 43, 68) RGC loss (13, 15, 27) Increased latency visual evoked potentials (9, 53, 77) Thinning in anterior regions of retina (RNFL and GCL + IPL) (15, 53) Outer neural retina areas (ONL; PR) varied responses after optic neuritis (15) GCL‐IPL thinning 1 month after optic neuritis (42)	RGC loss (8, 16, 59, 83, 84) OCT: Decreases in RNFL (21, 68, 76) ERG: Decreases in b‐wave and a‐waves (8, 24, 27, 32)
Optic nerve	Optic nerve atrophy correlated with whole‐field VEP amplitude (44, 54) Decrease in axonal densities and axonal loss (57, 70) Reduced axial diffusivity in optic nerve 1 month after optic neuritis (6)	
Optic tract	Optic tract lesion abnormalities correlated with RNFL thinning (48, 54) Decrease in axonal densities and axonal loss Optic Nerve damage (16, 39, 89)	
Lateral geniculate nucleus	Atrophy (11, 55, 74, 76, 81) Neuronal apoptosis after axon damage in optic tract (65)	Significant synapse loss at the LGN during early EAE (78)
Optic radiations	Lesions in the optic radiations (11, 76)	
Visual cortex	Atrophy associated with functional deficits in vision (11, 13, 20, 55)	

Review (citation 7 and 70): Balcer LJ, Miller DH, Reingold SC, Cohen JA (2015) Vision and vision‐related outcome measures in multiple sclerosis. Brain. 138(1):11–27. Pula JH, Reder AT (2009) Multiple sclerosis. Part I: neuro‐ophthalmic manifestations. Curr Opin Ophthalmol. 20(6):467–75.

EAE: 34, 66, 52.

Retina and optic nerves: 3, 19, 32, 72, 88.

LGN, optic radiations, and visual cortex in MS: 2, 31, 29, 62, 81.

VEPs: 11, 49, 71, 72.

Visual dysfunction: impaired contrast sensitivity, low‐contrast letter acuity, and color discrimination (19, 45, 54, 71) with source 7.

Rodent RGC neurons located in the GCL of the retina receive information from photoreceptors and send this information to the visual cortex through unmyelinated axons inside the retina that become myelinated once they reach the optic nerves. The optic nerves converge at the optic chiasm and continue as optic tracts. The optic tract axons cross at the midline to project contralaterally or ipsilaterally to the main mammalian visual targets: the LGN in the thalamus or the superior colliculus (SC). In humans, the distributions of these projections are distinct with individual eyes, which contribute projections to both the ipsilateral and contralateral LGN and SC. The LGN projects mainly to the primary visual cortex (V1) while the SC targets the thalamus and brainstem, providing two potential pathways for processing visual inputs. As the SC is primarily responsible for the efferent visual pathway, specifically eye movements, SC were not analyzed in the current study.

### 4.1 The retina

Patients with MS demonstrate progressive thinning in anterior regions of the retina, such as the RNFL and GCL + IPL (15). In medial areas of the retina, such as the INL and OPL, there is often no MS‐induced effect observed, although one study indicated a correlation between macular thinning, high expanded disability status scale scores, and thinning of the middle retina (57). Outer neural retina areas such as the ONL and PR can have varied responses after optic neuritis, depending on disease stage. Acute swelling of these layers often occurs within 1 month after ON onset, followed by either no discernable difference in thickness from baseline, or a progressive decline (15, 58).

Decreases in RGC numbers during EAE have been reported previously (16, 17, 59). Calpain inhibition and N‐Methyl‐D‐Aspartate receptor blockade has been shown to attenuate apoptosis of RGCs during acute ON (59, 60). In this study, IndCl treatment during EAE mitigated caspase‐3 in RGCs. It is well documented that calpain and caspase‐3 pathways cross talk and, caspase‐3 has been shown to cleave calpastatin (endogenous calpain inhibitor), which can result in the activation of calpain and the breakdown of target proteins (61). IndCl treatment was also shown to decrease caspase‐3 activity in OLs and increase cell survival. However, the increased RGC cells survival did not mitigate the decreases in RNFL layer by OCT, or ERG assessed amplitude and latency changes.

### 4.2 The optic nerve, optic chiasm, and optic tracts

These tracts are highly myelinated structures within the visual system comprised of RGC axons, OLs, microglia, and astrocytes. In MS patients, almost all white matter tracts of the afferent visual pathway are affected. The pathophysiology of permanent visual deficits in MS is recognized to be due to irreversible losses of myelin and axons with secondary neuronal degeneration (62). The optic nerve and OR are particularly prominent targets for demyelination (13, 20, 22). Optic nerve atrophy is correlated to thinning of the RNFL, macular volume loss, visual acuity, and whole field VEP amplitude, but not latency. Optic tract lesion abnormalities evident in MS patients are also correlated to RNFL thinning (63, 64) and decreases in axonal densities. The optic nerve and optic tract are heavily myelinated in the rodent. The present results revealed that the optic nerve and optic tract undergo significant inflammation, demyelination, astrogliosis, and axonal damage during EAE. IndCl treatment was able to attenuate inflammation and increase remyelination. Axon damage may be initiated in the early inflammatory period of MS and EAE, which can be identified with longitudinal diffusion tensor imaging (DTI) and VEPs, approaches that are currently being investigated (63, 64)

There is currently controversy on whether RGC loss and retinal pathology occur as a result of optic nerve damage, or whether retinal damage occurs separately and may contribute to optic nerve damage later (45, 65, 66). In contrast, GCL‐IPL thinning is evident within 1 month after ON, indicating that perhaps optic nerve stress may contribute to RGC death (67). In MS, longitudinal DTI studies show reduced axial diffusivity in the optic nerve 1 month after ON, indicating that axonal degradation preceded RNFL thinning in the affected eyes of early MS patients, both 6 and 12 months after ON (68). This suggests that optic nerve axon damage may precede RNFL loss (68). In either case it appears that there may be differing mechanisms driving RGC, RNFL, and optic nerve axon pathology.

### 4.3 The LGN

Atrophy of the LGN is correlated to lesions in the OR (21, 69), as well as thinning of the RNFL (21). Axon damage in the optic tract has been shown to cause significant atrophy and neuronal apoptosis in the LGN (70) of MS brains (62). Demyelination along with extensive inflammation and astrogliosis in the dLGN during EAE was not mitigated by treatment with IndCl. It is possible that extensive anterograde degradation of axons in the optic nerve and optic tract prior to treatment may have led to other pathologies besides demyelination, and treatment during peak EAE disease is too late. In fact, a recent paper showed a significant synapse loss at the LGN during early EAE (71).

### 4.4 The visual cortex

In MS the visual cortex undergoes significant atrophy, often associated with lesions or pathology of the OR, RNFL thinning, and functional deficits in vision (13, 20, 23, 69). Similarly, EAE‐induced demyelination and retraction of dendrites in the visual cortex were observed. Both were mitigated significantly in the presence of IndCl. Similar to what was seen in MS, visual cortex pathology was associated with RNFL thinning in the retina, upstream demyelination, and damage to visual pathway tracts.

### 4.5 OCT, ERGs, and VEPs

Decreases in RNFL have been seen in EAE by OCT (18, 72, 73). However, different labs have also shown varying changes in retinal layers via OCT. In these investigations, an OCT‐automated segmentation program, originally developed for human analysis, along with manual correction were used. These methods had the potential to bias retinal segmentation with increased experimental errors (18, 72, 73). The current study used automatic segmentation with Bioptigen Diver 3.0 software developed specifically for the mouse, then measured individual retinal layer thicknesses. This software segmented individual retina images and avoided the inclusion of blood vessel diameter in RNFL calculations. The results presented here were consistent over several experiments with error free, retinal layer thickness measures in normal and EAE retinas.

Even though OCT has become a widely used tool to assess neuronal and axonal degeneration in the retina, OCT does not measure retinal neuronal function. The function of the photoreceptors and bipolar cells can be measured using ERG electrophysiology. In the current study, EAE‐induced changes were similar to reports in MS, where there were decreases in the b‐wave, or both a‐ and b‐waves (74, 75, 76). In our study with EAE, a‐wave and b‐wave amplitudes were decreased, and latencies were increased. Significant changes to the photoreceptor layer have been shown to occur in the acute stages of ON with significant decreases in EAE a‐wave amplitudes compared to normal controls (15, 58, 77, 78). Inflammation of the retinal vasculature could lead to inner retinal dysfunction, while choroidal inflammation could be the cause of altered outer retinal function. Without further studies on EAE‐induced pathology in photoreceptor and RPE cells, we are unable to draw a precise conclusion as to the underlying etiology. Such studies are especially crucial because of the similar events observed in MS patients. Significant functional deficits were observed consistently during EAE using ERG and VEP analyses. Treatment with IndCl did not increase peak amplitude but did show a decrease in peak latencies as assayed with the VEP. Therapeutic treatment with IndCl was initiated after peak EAE disease when the mice already demonstrated significant motor deficits.

Past studies have shown that EAE‐induced inflammation in visual pathway white matter tracts occurs 7–8 dpi, demyelination 1–2 dpi, and axonal degeneration and neuron loss at 14 dpi (31, 79). Furthermore, neurodegenerative damage in visual pathway nuclei and tracts often has trans‐synaptic anterograde and retrograde effects. For instance, optic nerve transection in rodents not only results in upstream RGC death (80), but also causes apoptosis of synaptic targets in the LGN (70, 71). Similarly, ablation of a region in the visual cortex induces apoptosis of geniculocortical projection neurons (81, 82), while stroke‐induced visual cortex damage in humans causes progressive thinning of the RNFL (83). Thus, damage to any visual pathway area has significant upstream and downstream neurodegenerative effects. Optic nerve axon damage in EAE remained with IndCl treatment, indicating a possible breakdown of synaptic transmission to target nuclei such as the LGN and/or visual cortex. Given this disease profile, irrecoverable neuronal damage had likely occurred between the optic tract, LGN, and visual cortex prior to initiation of IndCl treatment. This may have contributed to the impaired VEP amplitudes observed even with IndCl treatment during EAE. The study results are modeled in Figure 8.

Although VEP amplitudes were not significantly improved with IndCl treatment, EAE‐induced increases in latency were abrogated to almost baseline levels, indicating functional remyelination. We have previously demonstrated the remyelinating capabilities of IndCl in the cuprizone and EAE mouse models of MS (40). Mice given 9 weeks of CPZ diet, sufficient to induce a 50% loss of myelin within the corpus callosum, showed robust myelination in response to IndCl treatment compared to vehicle controls. It is important to note that as IndCl and vehicle treatments were initiated after the CPZ demyelination period, these effects were a result of remyelination rather than protection from demyelination. Additionally, EAE mice treated with IndCl showed functional improvements in compound action potentials within the corpus callosum and spinal cord compared to vehicle controls. The known remyelinating capabilities of IndCl, coupled with the observed improvements in VEP latencies and visual pathway myelination suggest a similar remyelinating role for IndCl in the visual pathway.

Exactly how IndCl treatment may induce functional remyelination in the visual pathway requires further investigation. We have previously shown that although IndCl may not modify the extent of leukocytic infiltration into the CNS, it decreases the production of pro‐inflammatory cytokine interferon gamma (IFNγ) and CXCL10 by peripheral leukocytes, while increasing the production of anti‐inflammatory cytokine CXCL1 by astrocytes (37, 40). Intraocular injection of IFNγ increases latency measurements of VEPs while exerting subtle changes in myelinated visual system axons (84). It is possible that IndCl treatment attenuated IFNγ and CXCL10 production while promoting CXCL1 production within the visual system, thus mitigating further axonal demyelination and OL death, but may not be able to reverse the axon damage that occurred before treatment. Future studies are needed to investigate these potential mechanisms.

Studies which have assessed the capability of other therapeutics to attenuate functional deficits in VEPs have shown a lack of improvement with therapeutic treatment in animal models. Similar to IndCl (37, 40), the oral sphingosine 1‐phosphate receptor modulator, fingolimod, was shown to improve EAE‐mediated paralysis, decrease demyelination in the brain and spinal cord, and improve conduction in the CNS with therapeutic treatment (85). However, although prophylactic fingolimod improved VEP latencies, therapeutic treatment resulted in no identifiable recovery during EAE. Glatiramer acetate and Interferon β‐1b, other therapeutics currently approved for MS treatment, have also shown some improvement in VEPs during EAE. However, similar to fingolimod, these therapies were given prophylactically and thus lack clinical relevance. Conversely, therapeutic treatment with IndCl enhanced myelination in all structures of the visual pathway and caused a decrease in P1 and N2 latencies similar to that seen in over‐myelinating Akt overexpressing transgenic mice (86).

Studies in humans give conflicting reports on the efficacy of currently approved MS drugs for alleviating VEP deficits in MS patients. IFN β‐1b (87) and Natalizumab (88) attenuate visual dysfunction in the relapsing remitting form of MS. Some studies have shown improvements in visual function as measured with VEPs with Mitoxantrone (45) and 4‐aminopyridine treatment (89); however, it is unclear which patients in those studies had chronic progressive and which had relapsing remitting MS. Because the MOG‐EAE model used in the current study more closely recapitulates the chronic progressive disease profile, IndCl treatment has the potential to improve VEP latencies in chronic progressive MS, a result that has not been shown with current approved MS drugs.

### 4.6 The role of the ERβ ligand IndCl

While the current approved MS drugs attenuate inflammation, these drugs do not prevent neurodegeneration or initiate remyelination. In addition, these available immunomodulatory therapies do not stop the pathogenesis of MS and only partially prevent the onset of permanent disability in patients with MS. Due to this, there is a strong need to find a therapeutic candidate that restores neurological function in patients in this autoimmune, demyelinating disease. There is accumulating evidence that demonstrates the neuroprotective and immunomodulatory benefits of estrogens, making these possible candidates for MS treatment. This was demonstrated in preclinical studies where treatment with pregnancy levels of estriol attenuated the EAE disease severity (90, 91). The interest in utilizing ERβ ligands is also supported by results found from similar ligands such as diarylpropionitrile (DPN). IndCl is a halogen‐substituted phenyl‐2H‐indazole core with up to 100‐fold relative binding affinity for ERβ over ERα, which is important because the carcinogenic effects of estrogens are mediated through ERα (36, 92). The hope is that this ligand will be suitable for preclinical development and transition from bench to bedside.

Prophylactic and therapeutic IndCl treatment in EAE mice demonstrated decreased clinical disease severity scores with an improvement in myelination and neuroprotection in the spinal cord compared to vehicle treatment (40). In addition to this, IndCl improved function in demyelinated axons by improving rotarod performance and increasing the amplitude of compound action potential recordings (40).

It is important to note that ERβ is expressed in neurons, OLs, astrocytes, microglia, and immune cells (93). Previously, we have shown that ERβ is expressed in OL lineage cells (94). We conditionally knocked out ERβ in OL lineage cells which prevented an ERβ ligand (diarylpropionitrile)‐induced improvement in clinical disease and myelination, partially prevented callosal conduction improvement, and prevented activation of PI3K/Akt/mTOR pathway in callosal homogenates implicated in OL survival/axon myelination (94). Our results indicate that ERβ ligand‐conferred remyelination‐induced neuroprotection in EAE is mediated partially by ERβ in OLs. It is likely that in addition to acting directly on OLs to stimulate myelination, IndCl also acts on other cell types that express ERβ. While we have shown that ERβ is expressed on RGCs, we cannot directly conclude that IndCl is acting on this cell type exclusively without performing additional experiments. Previously, we have also shown that IndCl treatment accelerates remyelination in the chronic cuprizone model and decreases radial diffusivity by DTI, emphasizing a possibility for IndCl to improve myelination and function (95). ERβ ligands with structures similar to IndCl have also shown increased myelination and decreased neurodegeneration as well in the EAE animal model of MS (39).

### 4.7 Importance of investigating visual pathway dysfunction in MS and EAE

Acute demyelination is an important contributor to visual dysfunction in MS. The pathophysiology of permanent visual deficits is recognized to be due to irreversible losses of myelin and axons with secondary neuronal degeneration. In this study, we established a preclinical drug assessment paradigm capable of screening therapeutics for functional efficacy, remyelinating, and neuroprotective potential in the visual system. Because visual pathway dysfunction has significant detrimental effects to the quality of life experienced by MS patients, understanding the pathophysiology of the visual pathway in demyelinating disease is vital to therapeutic development. Furthermore, correlating minimally invasive functional assessments to pathological changes in the CNS during treatment has the potential to minimize drug discovery time and improve treatment procedures for patients. In this spirit, we assessed the efficacy of IndCl and demonstrated that IndCl attenuated visual system pathology and some functional deficits in EAE. Taken in conjunction with our past studies that show IndCl is a potent neuroprotective and remyelinating drug, the current work supports IndCl and ERβ ligands as exciting therapeutic agents for attenuating CNS pathology.

The cost of much‐needed research and clinical developments of effective, neuroprotective MS therapeutics is tremendous. Existing approved MS treatments delay, but fail to prevent or reverse, disease progression. How can we mitigate the cost of therapeutic candidate development while maximizing the number of drugs screened? The answer requires: (i) effective, representative preclinical animal models, and (ii) fully qualified surrogate endpoints that can be readily translated from animal models to human trials. EAE, one of the best models of MS, recapitulates inflammation, demyelination, and neurodegeneration components of the MS disease. However, current EAE endpoints used to assess therapeutic efficacy (e.g., limited spinal cord‐dependent clinical motor scores, invasive peripheral and CNS IHC) are not translationally realistic.

To obtain a meaningful functional recovery in demyelinating diseases, treatment should be started early, and these therapeutics must not only target remyelination, but must also prevent further axonal degeneration and neuronal loss (Figure 8). Because visual pathway dysfunction can be assessed longitudinally in a minimally invasive manner, studying ON in EAE is translationally relevant for understanding the onset and intensity of disease and screening better therapeutics for functional efficacy in the visual system.

## CONFLICT OF INTEREST

The authors declare that they have no conflict of interest.

## AUTHOR CONTRIBUTIONS

SKTW, JAK, and SN conceived and designed the experiments. MTS, KL, KCA, and BG performed the experiments, MTS, KL, KCA, MF, JSS, and CD analyzed the data, SHK and MC performed specific techniques and compounds. MTS, SKTW, KCA, and JAK wrote and edited the manuscript.

## Supporting information

Supplementary Material
**TABLE S1** Primary antibodies used for examining myelin, leukocytes, astrocytes, parvalbumin positive interneurons, RGCs, apoptosis and ERβ reactivity. Antibodies were paired with either Cy5 or Cy3 secondary antibodies for immunohistochemistry
**FIGURE S1** EAE retinas exhibit increased microglial and astrocytic activation which is attenuated with IndCl treatment. (A) Retina sections from groups shown in Figure 1A were collected 60 dpi and immunostained for Iba1 (green) and GFAP (red). Sections were imaged at 20x magnification with representative images shown. Iba1 and GFAP immunoreactivity is increased in the GCL, IPL, INL, and OPL in EAE + vehicle sections compared to normal and EAE + IndCl sections. (B) Percent area quantification shows increased Iba1 and GFAP. (C) immunoreactivity in retina sections from EAE + vehicle groups compared to normal and EAE + IndCl groups (n = 5). There was a decrease in GFAP immunoreactivity in the EAE + IndCl mice compared to EAE + vehicle. *p < 0.05, ***p < 0.001 ordinary one‐way ANOVA with Kruskal‐Wallis multiple comparison test. Scale bar is 100 µm
**FIGURE S2** Optic nerve axons synapse on neurons in the LGN, a major relay station which transmits information to higher order areas of the visual system. Consequently, RGC loss coupled with EAE pathology may have neurodegenerative effects on the LGN. (A) To investigate EAE‐mediated pathology and evaluate IndCl treatment effects in the LGN, Thy1‐YFP coronal sections containing LGN corresponding to plates 50 – 52 of the Paxinos and Franklin mouse brain atlas from normal, EAE + vehicle, and EAE + IndCl treated EAE male mice were collected 60 dpi and immunostained with CD45, GFAP, MBP, and parvalbumin (PV) antibodies and analyzed within the ventral LGN (B). (C) In vehicle, CD45 staining revealed large perivascular lesions surrounded by GFAP+ activated astrocytes which were absent in the normal group. (D and E) MBP staining intensity and the number of PV+ cells were depleted in vehicle tissues compared to normal. (E and J) The morphology of Thy1+ projection neurons was not noticeably changed between normal and vehicle groups, however, Thy1+ axons in vehicle tissues were fragmented and showed swollen regions that were not seen in normal, as indicated by the white arrows in the inset. (E and I) PV+ inhibitory interneurons were decreased in vehicle compared to normal. (F and G) Quantification of CD45+ leukocytic and GFAP+ astrocytic intensity showed significant increases in vehicle compared to normal. (H) Tissues from EAE + vehicle mice showed a significant loss of MBP intensity compared to that seen in normal tissues. (J) EAE + vehicle tissues also exhibited a significant decrease in the number of Thy1+ axon swellings compared to normal

## Data Availability

The data that support the findings of this study are available from the corresponding author upon reasonable request.
