# Supplementary material for: Alleviation of extensive visual pathway dysfunction by a remyelinating drug in a chronic mouse model of multiple sclerosis
Source: Brain Pathol. 2021 Jan 29;31(2):312–32. doi: 10.1111/bpa.12930 (PMC8018057; doi:10.1111/bpa.12930)
Supplement: Supplementary file 1 — Supplementary Material TABLE S1 Primary antibodies used for examining myelin, leukocytes, astrocytes, parvalbumin positive interneurons, RGCs, apoptosis and ERβ reactivity. Antibodies were paired with either Cy5 or Cy3 secondary antibodies for immunohistochemistry FIGURE S1 EAE retinas exhibit increased microglial and astrocytic activation which is attenuated with IndCl treatment. (A) Retina sections from groups shown in Figure 1A were collected 60 dpi and immunostained for Iba1 (green) and GFAP (red). Sections were imaged at 20x magnification with representative images shown. Iba1 and GFAP immunoreactivity is increased in the GCL, IPL, INL, and OPL in EAE + vehicle sections compared to normal and EAE + IndCl sections. (B) Percent area quantification shows increased Iba1 and GFAP. (C) immunoreactivity in retina sections from EAE + vehicle groups compared to normal and EAE + IndCl groups (n = 5). There was a decrease in GFAP immunoreactivity in the EAE + IndCl mice compared to EAE + vehicle. *p < 0.05, ***p < 0.001 ordinary one‐way ANOVA with Kruskal‐Wallis multiple comparison test. Scale bar is 100 µm FIGURE S2 Optic nerve axons synapse on neurons in the LGN, a major relay station which transmits information to higher order areas of the visual system. Consequently, RGC loss coupled with EAE pathology may have neurodegenerative effects on the LGN. (A) To investigate EAE‐mediated pathology and evaluate IndCl treatment effects in the LGN, Thy1‐YFP coronal sections containing LGN corresponding to plates 50 – 52 of the Paxinos and Franklin mouse brain atlas from normal, EAE + vehicle, and EAE + IndCl treated EAE male mice were collected 60 dpi and immunostained with CD45, GFAP, MBP, and parvalbumin (PV) antibodies and analyzed within the ventral LGN (B). (C) In vehicle, CD45 staining revealed large perivascular lesions surrounded by GFAP+ activated astrocytes which were absent in the normal group. (D and E) MBP staining intensity and the number of PV+ cells were depleted [file BPA-31-312-s001.docx]

**Supplementary Materials**

**A. Supplementary Methods**

**EAE Induction**:

Thy1-YFP transgenic mice, which express green fluorescent protein under a neuron-specific promoter of the Thy1 gene, were used to investigate EAE-induced effects on RGCs, optic nerve, optic tract axons, and visual cortex pyramidal neurons (26, 87). PLP-EGFP transgenic mice, which express green fluorescent protein under the OL specific proteolipid protein (PLP) promoter, were used to assay changes in number of OLs and myelin (66).

Mice were anesthetized with 2% isoflurane gas in oxygen at a flow rate of 2 L/min. On the first day of induction (Day 0), 0.05 ml of the emulsion was injected subcutaneously into the backs of the animals between the cervical vertebrae and left shoulder and another 0.05 ml between the lumbar vertebrae and left thigh. Injections were placed as such to maximize proximity to axillary and inguinal lymph nodes. Each mouse was then injected intraperitoneally with 0.3 ml of Pertussis toxin (PTx; List Biological Laboratories) in DPBS at 1.667 µg/ml, returned to its home cage with free access to water and food, and monitored until recovered. Two days after induction (day 2), the mice were given booster IP injections of pertussis toxin at the same concentration. Five days later (day 7), motor function was assessed using clinical scores, then mice were injected with a freshly made MOG_35-55_ /M. tuberculosis emulsion at the same ratios and concentrations used on Day 0 (Figure 1Aii, Bi). Day 7 MOG_35-55_ injections of 0.05 ml each were placed between the cervical vertebrae and right shoulder, and the lumbar vertebrae and right thigh.

**EAE Clinical Scoring and IndCl Treatment:**

Starting 7 days post induction (dpi), mice were scored daily for clinical disease severity. The clinical scoring protocol was defined as: 0, unaffected; 1, complete tail limpness; 2 failure to right upon attempt to roll over; 3, partial hind limb paralysis; 4, complete hind limb paralysis; and 5, moribund (45). Mice reaching clinical scores of 4.5 or higher were euthanized humanely with isoflurane anesthesia followed by cervical dislocation.

EAE disease onset occurred 10 to 13 dpi, reached a peak disease severity between 16 and 20 dpi, and maintained severity through late EAE and euthanasia at 60 dpi (Figure 1B, C).

**Optical Coherence Tomography:**

Animals were anesthetized with intraperitoneal injections of 100 mg/kg body weight ketamine (Putney Veterinary Generics) and 10 mg/kg bodyweight xylazine (Lloyd Laboratories) resulting in 1 to 1.5 hours of sedation. After an animal was fully sedated, eyes were dilated with 1% tropicamide (Alcon Laboratories) for 10 minutes. The animal was then placed on the OCT system and arranged such that the temporal field of the retina was in full view. Systane ultra-lubricant eye drops were applied to eye spears (Novartis) and applied intermittently throughout the procedure to prevent dehydration of the eye and cataract formation. One thousand A-scans and one hundred B-scans were taken to generate each OCT image. Each image was taken 3 times and averaged. After imaging, the eye was lubricated with ophthalmic ointment (Dechra Veterinary Products) to prevent cataract formation during the remaining anesthesia period (119). Post-experiment care included animals being placed in a large open-air container with a heating pad set to 40˚C and monitored. Once they awoke, they were returned to their respective home cages.

**Electroretinograms and Visual Evoked Potentials:**

Animals were anesthetized with isoflurane (2%; Piramal Healthcare), in sterile medical grade oxygen delivered through an isoflurane/oxygen induction chamber and/or face mask. Body temperature was maintained at 37°C. Eyes were dilated with 1% tropicamide for 10 minutes before recording and visine dry eye drops were applied every 2-3 minutes to hydrate eyes and prevent cataract formation (119).

Stainless steel subdermal electrodes (F-Needle Electrode (F-E2); OcuScience) were inserted at the base of the tail (ground electrode), and on either side of the snout (reference electrodes). For ERG recordings silver-embedded thread electrodes (1.5" Filament (2); OcuScience) were placed over the cornea and held in place with mini contact lenses filled with a saline solution to optimize conductivity between the electrode and the cornea. During VEP recordings subdermal electrodes were inserted 2-3 mm lateral to the midline above the visual cortex on the left and right side (46, 124). Mini contact lenses filled with saline solution were placed over the eyes during VEP recordings to prevent dehydration.

**Perfusions and Tissue Preparation**

Eyes, optic nerves, and brain were dissected and post-fixed in 10% formalin for 2 hrs. Then brain and optic nerve excluding eyes was transferred to 30% sucrose with 0.2% sodium azide for 2 days (Fisher Scientific) for cryoprotection. Tissue was embedded in a gelatin/sucrose solution (7.5% w/v gelatin (Becton Dickinson) +15% w/v sucrose (Fisher Scientific) in Milli-Q water. Gelatin tissue blocks were placed in 10% formalin overnight, followed by 30% sucrose + 0.2% sodium azide solution until ready for sectioning. Retina processing was performed by removing cornea and lens from eyes. Retina attached to sclera was transferred to 10% formalin for 24 hours followed by 30% sucrose + 0.2% sodium azide solution. Retinas were embedded in optimal cutting temperature compound, then placed in 2-methylbutane (EMD Millipore) on dry ice to allow for uniform freezing. Embedded retinas and optic nerve gelatin blocks were cut into 10 µm thick sagittal sections using a cryostat and affixed directly to warm slides. Brain gelatin blocks were flash frozen on dry ice, and cut into 40 µm thick coronal sections, then placed in PBS with 0.2% sodium azide.

**Immunohistochemistry**

Prior to antibody application, tissue sections were thoroughly washed with PBS to remove residual sodium azide, permeabilized with 0.3% Triton-X (Electron Microscopy Sciences) and blocked in 20% normal goat serum (NGS) (Sigma-Aldrich). Sections were incubated in 1:500 dilutions of primary antibodies (Table 1) for 2 hours at room temperature followed by overnight at 4^o^C. Following day, the sections were washed with PBS and then Tris buffered saline (TBS). The sections were incubated with the corresponding secondary antibodies: Goat anti-rabbit IgG Cy3 (EMD Millipore), Goat anti-Rat IgG Cy5 (Invitrogen), Goat anti-mouse Cy5 IgG (Invitrogen). Sections were co-stained with 4’,6-Diamidino-2-Phenylindole (DAPI; EMD Millipore) to quantify cell numbers. Finally, the sections were washed again, mounted, cover slipped.

Table 1. Primary antibodies used for examining myelin, leukocytes, astrocytes, parvalbumin positive interneurons, RGCs, apoptosis and ERβ reactivity. Antibodies were paired with either Cy5 or Cy3 secondary antibodies for immunohistochemistry.

| Antibody | Target | Vendor | Catalog # |
| --- | --- | --- | --- |
| MBP | Myelin basic protein | Abcam | ab40390 |
| CD45 | Cluster of differentiation 45; Pan leukocytic antigen (clone 30-F11) | Becton Dickinson | 550539 |
| GFAP | Glial fibrillary acidic protein | Invitrogen | 180063 |
| Iba-1 | Ionized calcium binding adaptor molecule 1 | Wako | 019-19741 |
| PV | Parvalbumin (clone PARV-19) | EMD Millipore | MAB1572 |
| RBPMS | RNA binding protein with multiple splicing | PhosphoSolutions | 1830 |
| Casp-3 | Caspase-3 (clone AM1.31-11) | EMD Millipore | AM65 |
| ERβ | Estrogen Receptor β | EMD Millipore | 05-824 |

**Microscopy, Quantification, and Statistics**

Immunofluorescence intensity and cell numbers were assessed with NIH ImageJ software (v1. 50i http://rsb.info.nih.gov/ij/) and quantified for CD45, MBP, and GFAP immunofluorescence, or cell counts for RBPMS+, Caspase-3+, ERβ+, PV+, Thy1-YFP+ and PLP-EGFP+ cells. Histograms were adjusted evenly to match those of control images and saved. Files were then converted to RGB tagged image format (.tif) files and transferred to ImageJ where individual color channels were converted to gray scale. Brightness and contrast were automatically adjusted to limit experimenter bias and a region of interest was traced around an area encompassing the retina, optic nerve, optic tract, LGN, or visual cortex. The threshold of staining for each image and thus the percentage of fluorescent pixels in each area of interest was computed by the software and reported in a table, which was transferred to Graphpad Prism (La Jolla CA) for statistical analysis. For cell counts in the retina, the images were taken at 40x and all relevant cells in the ganglion cell layer (GCL) were counted, corresponding to a length of 0.25 mm. In the optic nerve, PLP images were taken at 10x, GFAP and MBP and 20x, and Thy1 at 40x. For PLP images, PLP+ cells were counted across the whole image corresponding to an area of approximately 0.3mm2. Brain sections containing the optic tract were imaged at 20x, and LGN sections at 40x magnification. Visual cortex MBP sections were imaged at 10x, and Thy1 + PV sections at 20x. For Thy1 and PV cell counts, an overlay grid with bin dimensions of 316.23 x 316.23 pixels was placed over images and cells were counted in two or three of the grid areas respectively, depending on the density of cells in the image. These areas corresponded to a total area of approximately 0.02 or 0.03mm^2^, respectively. For axon swelling counts in white matter tracts, single-channel images from Thy1 sections were thresholded in the region of interest corresponding to the optic nerve or optic tract. The ImageJ analyze particles function was used to count the number of axonal blebs (particles) present in the thresholded image, within the region of interest. Results from all counts were analyzed in GraphPad Prism as well.

**Statistics:**

For immunohistochemistry, two sections per mouse were taken for each area of interest in the brain and three sections per mouse for retina and optic nerve tissues. There were 6-10 mice per treatment groups. For ERG, VEP, and OCT in vivo studies n=5-8 mice per group were used. Both eyes were assessed in all in vivo studies resulting in a minimum of n=10 eyes per group.

Statistics were performed using Prism (Prism®, GraphPad) program for Windows. Graph values are expressed as mean ± standard error of the mean. For histology, electron microscopy (EM), and in vivo studies statistical analysis of mean values was carried out using one-way ANOVA if mean values passed a normality test, or Kruskal Wallis multiple comparison test if they did not. For EAE clinical scores, statistics were performed using an ordinary two-way ANOVA with Bonferroni post-hoc test as previously described (45). Differences were considered significant at the *p<0.05, **p<0.01, ***p<0.001, and ****p<0.0001 level.

**B. Supplementary Figures:**

**
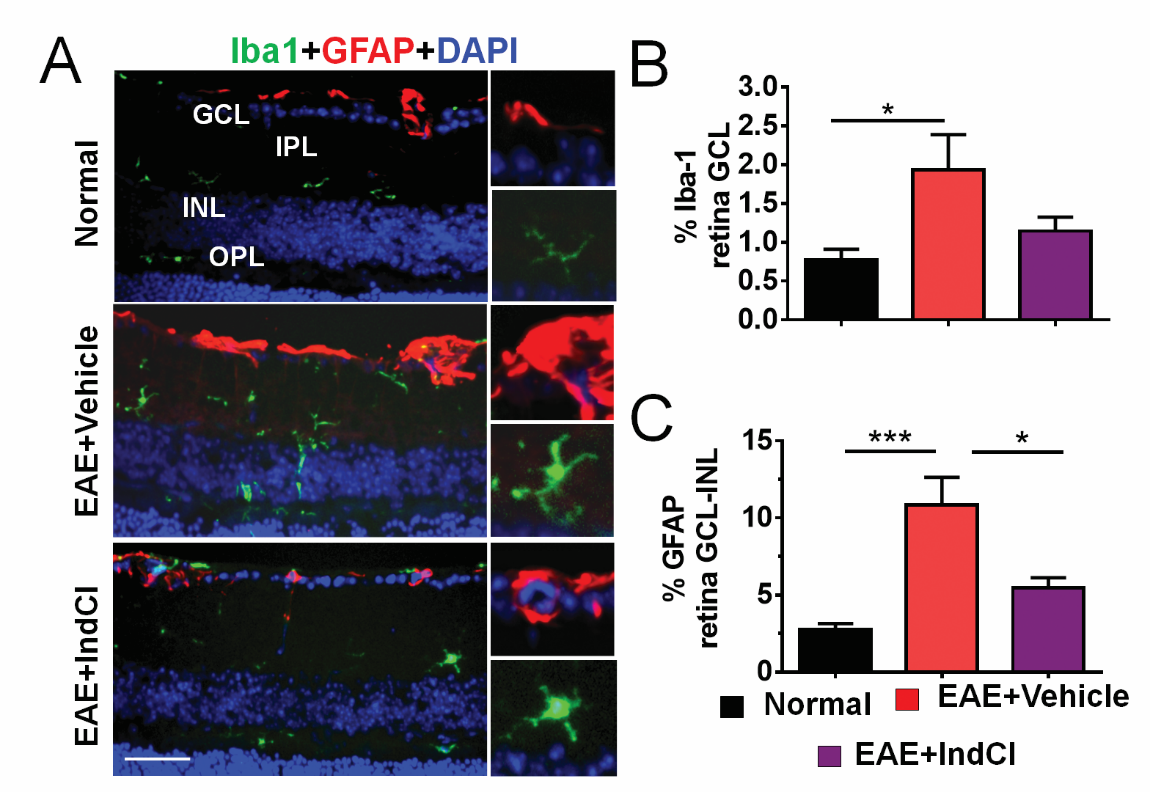
**

**Supplementary Figure 1: EAE retinas exhibit increased microglial and astrocytic activation which is attenuated with IndCl treatment. A.** Retina sections from groups shown in Figure 1A were collected 60 dpi and immunostained for Iba1 (green) and GFAP (red). Sections were imaged at 20x magnification with representative images shown. Iba1 and GFAP immunoreactivity is increased in the GCL, IPL, INL, and OPL in EAE + vehicle sections compared to normal and EAE + IndCl sections. **B.** Percent area quantification shows increased Iba1 and GFAP **C**. immunoreactivity in retina sections from EAE + vehicle groups compared to normal and EAE + IndCl groups (n=5). There was a decrease in GFAP immunoreactivity in the EAE + IndCl mice compared to EAE + vehicle. **P*<0.05, ****P*<0.001 ordinary one-way ANOVA with Kruskal-Wallis multiple comparison test. Scale bar is 100 µm.


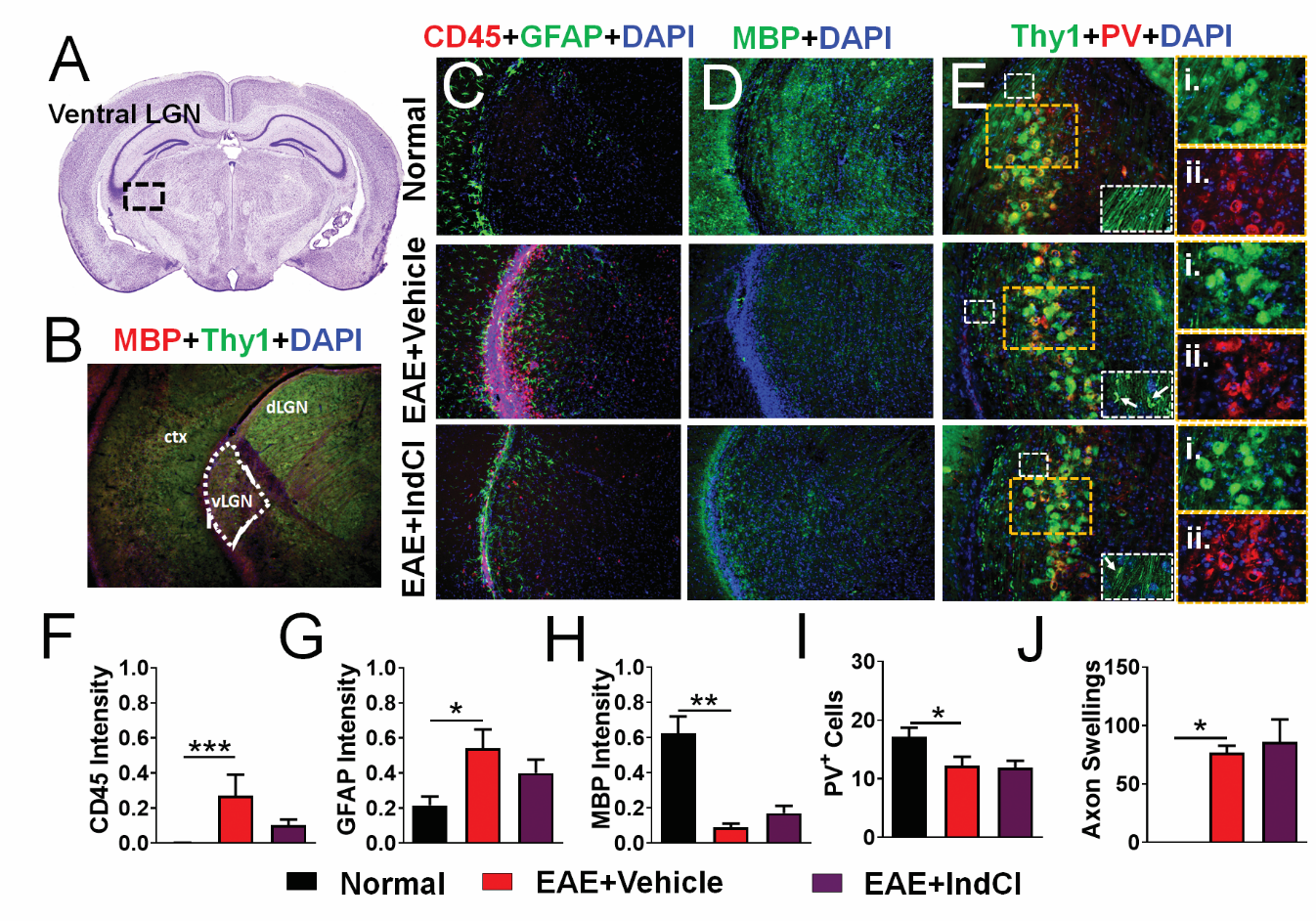


**Supplemental Figure 2:** Optic nerve axons synapse on neurons in the LGN, a major relay station which transmits information to higher order areas of the visual system. Consequently, RGC loss coupled with EAE pathology may have neurodegenerative effects on the LGN. **A.** To investigate EAE-mediated pathology and evaluate IndCl treatment effects in the LGN, Thy1-YFP coronal sections containing LGN corresponding to plates 50 – 52 of the Paxinos and Franklin mouse brain atlas from normal, EAE + vehicle, and EAE + IndCl treated EAE male mice were collected 60 dpi and immunostained with CD45, GFAP, MBP, and parvalbumin (PV) antibodies and analyzed within the ventral LGN **(B)**. **C.** In vehicle, CD45 staining revealed large perivascular lesions surrounded by GFAP+ activated astrocytes which were absent in the normal group. **D,E.** MBP staining intensity and the number of PV+ cells were depleted in vehicle tissues compared to normal. **E,J.** The morphology of Thy1+ projection neurons was not noticeably changed between normal and vehicle groups, however, Thy1+ axons in vehicle tissues were fragmented and showed swollen regions that were not seen in normal, as indicated by the white arrows in the inset. **E,I.** PV+ inhibitory interneurons were decreased in vehicle compared to normal. **F,G.** Quantification of CD45+ leukocytic and GFAP+ astrocytic intensity showed significant increases in vehicle compared to normal. **H.** Tissues from EAE + vehicle mice showed a significant loss of MBP intensity compared to that seen in normal tissues. **J.** EAE + vehicle tissues also exhibited a significant decrease in the number of Thy1+ axon swellings compared to normal.
